# The total extract of *Abelmoschus manihot* (L.) medic flowers (TEA) mediated Nrf2-TFAM signalling to regulate mitochondrial antioxidant mechanism

**DOI:** 10.1038/s41598-024-84022-x

**Published:** 2025-01-10

**Authors:** Ying Song, Xinyi Zhu, Beibei Wang, Qisong Li, Biwei Song

**Affiliations:** 1 Department of Pharmacology, Zhejiang University of Technology, 18 Chao-Wang Road, Hangzhou, 310014 Zhejiang People’s Republic of China; 2 Department of Pharmacy, Huzhou Maternity & Child Health Care Hospital, Huzhou, 313000 Zhejiang People’s Republic of China; 3 Hangzhou King’s Bio-Pharmaceutical Technology Co., Ltd., Hangzhou, 310007 Zhejiang People’s Republic of China

**Keywords:** Nrf2, TFAM, Oxidative stress, Mitochondria, The total extract of *Abelmoschus manihot* (L.) Medic flowers (TEA), Cell biology, Molecular biology

## Abstract

Skin, as the first line of defence of the human body, is exposed to dangers such as overheating substances, ultraviolet rays, and environmental pollutants, and the incidence of skin diseases is increasing annually. Oxidative stress plays a dominant role in most skin diseases. *Abelmoschus manihot* (L.) medic flower (TEA) is a traditional Chinese medicine widely used to treat injuries to the skin such as water and fire scalds. It has been reported that TEA has excellent antioxidant effects. In this study, we aimed to explore the antioxidant and mitochondrial protection effects of TEA in H_2_O_2_-mediated HaCaT cell damage. HaCaT cells were incubated with H_2_O_2_ to simulate oxidative stress in the skin. The effect of TEA on HaCaT cells was also evaluated. Cell morphology was observed via inverted microscopy, and cell viability was measured via the MTT reagent. The cells were stained with Hoechst 33,324 solution. Reactive oxygen species (ROS), superoxide dismutase (SOD), malondialdehyde (MDA) and ATP detection kits were used to detect the corresponding indicators. The mitochondrial membrane potential was detected by JC-1. RT-PCR was used to detect mRNA and mtDNA expression. The expression of the target protein was detected by Western blotting and immunofluorescence. H_2_O_2_ triggered oxidative damage in HaCaT cells, which manifested as apoptosis, increased ROS and MDA contents, and decreased SOD activity. H_2_O_2_ activates the KEAP1/Nrf2/NQO1 signalling pathway, which decreases the expression of the intracellular KEAP1 protein and slightly increases the expression of the Nrf2 and NQO1 proteins, further causing mitochondrial oxidative stress, resulting in changes in the mitochondrial membrane potential, a reduction in the mtDNA copy number, and decreased expression of the PGC-1α and TFAM proteins. In addition the expression of mitochondrial respiratory chain genes and proteins decreased. TEA promoted the expression of Nrf2 in HaCaT cells, activated the downstream antioxidant response, and alleviated the oxidative stress and mitochondrial damage caused by H_2_O_2_. ML385 is an Nrf2 inhibitor, under which the antioxidant and mitochondrial protective effects of TEA are inhibited. When TFAM was knocked down, the protective effect of TEA on mitochondria was also inhibited. TEA protects HaCaT cells from H_2_O_2_-induced oxidative damage and mitochondrial oxidative damage through the KEAP1/Nrf2/NQO1/PGC-1α/TFAM pathway.

## Introduction

The skin is a multifunctional organ system that plays a vital role in physiological homeostasis, including thermoregulation, maintaining fluid balance, and preventing pathogens. As the body’s first defence barrier, the skin is constantly exposed to external stimuli such as ultraviolet radiation (UVR)^1^, environmental pollutants^2^, industrial chemicals^3^, and high-temperature hazardous materials^4,5^. These stimuli can induce the production of reactive oxygen species (ROS), thus mediating oxidative stress (OS)^6,7^.

Many reports^8,9^ have shown that skin burns can cause OS, and a large number of reactive oxygen species accumulate in the skin to further aggravate the injury of the burn wound, causing oedema, inflammatory reactions, damage to other organs of the body, such as the lungs, the kidneys and the livers damage, and even shock. Chen et al.^10^ simulated heat stress with H_2_O_2_ in vitro and reported that hypoxia-induced factor 1 (HIF1) regulated oxidative stress-induced apoptosis in epidermal HaCaT cells by regulating the HIF1 signalling pathway. Long-term exposure to UVR^11^ leads to elevated nicotinamide adenine dinucleotide phosphate (NADPH) oxidase and ROS production, which increases inflammation, cytokine, chemokine, and skin ageing. Chronic and persistent inflammation caused by UVR weakens the skin’s defence mechanisms, degrades collagen and elastin fibers, and ultimately leads to premature aging. Vitamin C^12^ was found to have protective effects on UVB-induced oxidative stress, DNA modification and double-strand breaks in keratinocytes. In addition, OS has been found in psoriasis^12^, specific dermatitis and other diseases. Alleviating skin oxidative stress and mitochondrial oxidative stress is the key to treatimg of many skin diseases.

Abelmoschus manihot (L.) Medic flower (*AM*) is a traditional Chinese medicine used for treating skin diseases such as burns and carbuncles. The main chemical components of *AM* are flavonoids, including hyperin, isoquercetin, quercetin-3-O-glucoside, rutin, myricetin and quercetin^13^. At present, research on *AM* has focused mainly on its antioxidant and anti-inflammatory effects on liver and kidney diseases and gastric ulcers. For example, Zhang et al.^14^ rreported that total flavonoids extracted from *AM* alleviated acute ulcerative colitis via antioxidant activity and blocked TNF-α-induced NF-κB activation, which is consistent with the finding of Xue et al.^15^. Yan et al.^16^ found that *AM* extract can protect mice from D-galactose-induced oxidative stress, which is related to the activation of Nrf2 signalling. At present, there are few studies on *AM* in skincare. Wang et al.^17^ found that the polysaccharides extract from the roots of *AM* had moisturizing, antioxidant, anti-radical and anti-aging activities in their preliminary investigation.

In this study, we explored the antioxidant effect of the total extract from the flowers of *AM* (TEA) on the skin and the specific mechanism of action. We used an in vitro model of H_2_O_2_-induced HaCaT cell damage^5,10^ to investigate the effect of TEA on epidermal oxidative damage and the underlying molecular mechanism. The hypothesis of this study was that TEA has an antioxidative effect on the skin through the Nrf2-TFAM pathway.

## Materials and methods

### Cell culture study

Normal human immortal epidermal HaCaT cells were obtained from the Peking Union Cell Resource Center and cultured in minimum essential medium (MEM, Procell, China) supplemented with 100 U/ml penicillin G and 100 mg/ml streptomycin (Procell, China). China) and 15% foetal bovine serum (FBS, Gibco, USA). HaCaT cells were incubated at 37 °C in an incubator containing 5% CO_2_. During H_2_O_2_ treatment, different doses of H_2_O_2_ (0, 50, 75, 100, 125 and 150 µM) were added to complete cell medium and cultured for 24 h.

To construct HaCaT cell lines with stable low expression of TFAM, 1.5 × 10^5^–2.0 × 10^5^ cells were inoculated in a 6-well culture plate containing a standard growth medium without antibiotics 24 h before transfection, and the cells were allowed to grow to a confluence of 40–60%. An appropriate amount of venom (Haoji, Hangzhou, China) was added, and the mixture was incubated at 37  C overnight. The TFAM gene target sequence: 5 '-GTAAGTTCTTACCTTCGATTT-3'. Puromycin was used to screen HaCaT cell lines downregulated by TFAM gene. After screening, the expression of TFAM was detected via real-time quantitative PCR and Western blotting.

### Preparation and detection of TEA

TEA was prepared via the ethanol reflux method^5^. Dried *AM* was purchased from Bozhou Yuanshengtang Pharmaceutical Co., Ltd., Anhui Province. A total of 318.8572 g of *AM* powder was crushed and screened, and 10 volumes of 80% ethanol were added. The *AM* powder was repeatedly extracted by reflux heating 3 times for 2 h each time. After filtration, the mixture was mixed with the filtrate, and concentrated under reduced pressure via a rotary evaporator to obtain concentrated TEA. The specific methods used for sample identification and sample concentration determination are described in the supplementary materials.

### Reagents and antibodies

Primary antibodies against Nrf2 (lot no.16369-1-Ig), TFAM (lot no.22586-1-AP), KEAP1 (lot no.60027-1-Ig), PGC-1α (lot no.66369-1-Ig), Nrf1 (lot no.66832-1-Ig), CYTB (lot no.55090-1-AP) and GAPDH (lot no.60004-1-Ig) were purchased from Proteintech (Wuhan, China). Primary antibodies against NQO1 (lot no.D261049), MTCO1 (lot no.D163924), and MT-ATP6 (lot no.D10058) were purchased from Sangon (Shanghai, China). Horseradish peroxidase-conjugated anti-rabbit IgG and horseradish peroxidase-conjugated anti-mouse IgG were purchased from Biosharp (Shanghai, China).

### Assessment of cell viability with MTT

HaCaT cells were seeded at a density of 6,000 cells/well in 96-well plates and cultured for 24 h. After that, different concentrations of H_2_0_2_ (0, 50, 75, 100, 125 and 150 µM) were added to the complete MEM culture medium with 6 replicates per concentration. After culture for another 24 h, the culture medium was removed, and the residues were washed away with PBS. Each well was then replenished with 100 µl of MEM incomplete culture medium and 10 µl of a 5 mg/ml MTT solution, and the mixture was incubated in a culture box for 4 h. After the culture medium was removed, 150 µl of DMSO was added to each well to dissolve the blue crystals completely, and the absorbance of each ssample was measured at 570 nm via a microplate reader (SGNERGY H1, BioTek, America, in normal use). The above procedure was performed to determine the optimal modelling concentration of H_2_O_2_.

The effects of different concentrations of TEA (0, 3.125, 6.25, 12.5, 25, 50, 100 and 200 µg/ml) and VC (0, 3.125, 6.25, 12.5, 25 and 50 µg/ml) on the viability of HaCaT cells were examined using the same method. The above procedure was performed to determine the safe concentrations of TEA and VC.

After determining the optimal concentration of H_2_O_2,_ the activity of HaCaT cells subjected to oxidative damage caused by the optimal concentration of H_2_O_2_ with different concentrations of TEA (0, 3.125, 6.25, 12.5, 25 and 50 µg/ml) and VC (0, 3.125, 6.25, 12.5, 25 and 50 µg/ml) was detected via the same method. The above procedure was performed to determine the optimal concentration of TEA and VC for use in further experiments.

### Hoechst 33,342 staining

Hoechst 33,342 staining solution was added to cells seeded in 24-well plates, which were subsequently incubated at 37 °C in the dark for 5 min. The staining solution was subsequently removed, and the cells were observed under a fluorescence microscope (AX/AX R, Nikon, Japan, in normal use).

### Biochemical analysis

ROS content detection: HaCaT cells were seeded in 6-cm culture dishes. When the confluence rate reached 70–80%, the HacaT cells were treated with H_2_O_2_ (150 µM), H_2_O_2_ (150 µM) + HSK (3.125 µg/ml), or H_2_O_2_ (150 µM) + VC (3.125 µg/ml). After 24 h of drug administration, the cells were placed on ice, the culture medium was removed, and the samples were rinsed three times with precooled PBS. One millilitre of PBS was added to the culture dish, and the cells were scraped with a cell scraper and collected in a centrifuge tube. The collected cell suspension was split in half, and centrifuged at 3000 r for 5 min. One aliquot of cells was supplemented with 1 mM DCFH-DA (Beyotime) and incubated at 37  C in the dark for 30 min. The fluorescence intensity was detected via a fluorescence microplate reader with an excitation wavelength of 488 nm and an emission wavelength of 525 nm. Then, 50 µl of cell lysate (Beyotime) was added to another fraction of cells, which were subsequently lysed on ice for 15 min, scraped with a cell scraper and collected in a centrifuge tube. The supernatant was obtained by centrifugation at 12,000 × g for 10 min at 4 °C. The protein concentration of the supernatant was determined via a BCA kit (Bode). The relative ROS content was obtained by dividing the fluorescence intensity by the protein concentration.

SOD activity detection: Sample preparation: (1) Cell collection: Cell lysis buffer was added to the cells and they were lysed on ice for 15 min. The cells were collected in centrifuge tubes and centrifuged at 4 °C, and 14,000 × g for 10 min. The supernatant obtained by centrifugation was the sample to be tested. (2) Protein determination: A BCA kit was used to determine the protein concentration. SOD activit was determined according to the instructions of the superoxide dismutase (SOD) assay kit (WST-1 method) (A001-3-2, Nanjing Institute of Bioengineering, Nanjing, China), and was normalized to the protein concentration.

MDA content detection: The sample preparation was the same as that for the SOD assay, and the MDA content was determined according to the instructions of malondialdehyde (MDA) assay kit (TBA method) (A003-1-2, Nanjing Institute of Bioengineering, Nanjing, China), and normalized to the protein concentration.

ATP content detection: ATP lysis was added to the cells, which were then centrifuged at 4 °C 12,000 × g for 5 min, after which the supernatant was collected for subsequent assays. The ATP content in HaCaT cells was determined according to the instructions of the Enhanced ATP Assay Kit (Beyotime, Shanghai, China) and normalized to the protein concentration.

### JC-1 staining

An enhanced mitochondrial membrane potential assay kit with JC-1 (Beyotime, Shanghai, China) was used to detect changes in the mitochondrial membrane potential. One millilitre of JC-1 staining solution was added to cells seeded in a 6-well plate and incubated at 37 °C for 20 min. After incubation, the cells were washed twice with JC-1 staining buffer, cell culture medium was added, and the cells were observed under a fluorescence microscope.

### Quantitative real time-PCR and mtDNA content analysis

Total RNA was extracted from cells TRIzol (Invitrogen, USA, 12183-555) and reverse transcription was performed using a kit (Invitrogen, USA, 11752-050). The gene expression of related mRNAs was detected via the comparative cyclic threshold method with a 7500 real-time fluorescent quantitative PCR system (Thermo Fisher, USA, in normal use). The primer sequences (Sangon, Shanghai, China) are shown in Table 1. The data were normalized to the 18S rRNA mRNA level.

**Table 1:** Quantitative real time-PCR primers.

Gene	Primer sequence (5' to 3’)
Nrf2	F: ATAGCTGAGCCCAGTATC
	R: CATGCACGTGAGTGCTCT
TFAM	F: TTTCTCCGAAGCATGTGGGG
	R: CTTCAGCTTTTCCTGCGGTG
mtND3	F: ACCACAACTCAACGGCTACA
	R: TTATGGAGAAAGGGACGCGG
mtATP6	F: GGGCGCAGTGATTATAGGCT
	R: TAAGGGGTGTAGGTGTGCCT
mtCox5b	F: GGACAATACCAGCGTCGTCT
	R: TTGTAATGGGCTCCACAGCG
mtCyb	F: AACTTCGGCTCACTCCTTGG
	R: GGAGGTGATTCCTAGGGGGT
mtCO1	F: TTCGCCGACCGTTGACTATT
	R: TTGTTTATGCGGGGAAACGC
D-loop	F: AAGTGGCTGTGCAGACATTC
	R: TCTGTCTTTGATTCCTGCCT
18S rRNA	F: GGCCCTGTAATTGGAATGAGTC
	R: CCAAGATCCAACTACGAGCTT

To analyse the mitochondrial DNA (mtDNA) content, total DNA was extracted from cells using the Universal Genomic DNA Kit (CW2298S, CWBIO, Beijing, China) and 10 ng of DNA was subjected to qPCR analysis. The mtDNA copy number was measured via the mitochondrial D-LOOP gene and normalized to 18S rRNA. The primer sequences (Sangon, Shanghai, China) are shown in Table 1.

### Western blotting (WB)

The cells were washed 3 times with prechilled PBS, an appropriate amount of RIPA lysis buffer was added to the cells, and the cells were lysed on ice for 15 min. The mixture was centrifuged at 12,000 rcf, and the supernatant was collected as the protein sample.The protein concentration was determined via a BCA protein concentration assay kit (Boster, China) according to the manufacturer’s instructions. Forty micrograms of protein were subjected to a sodium dodecyl sulfate–polyacrylamide gel electrophoresis (SDS-PAGE), separated by electrophoresis, and transferred to a polyvinylidene fluoride (PVDF) membrane. The PVDF membrane with 5% skim milk at room temperature. After washing with TBST, the PVDF membrane was incubated with the primary antibody overnight at 4  C. After washing with TBST, the PVDF membrane was incubated with horseradish peroxidase-conjugated anti-rabbit or rat IgG for 1 h at room temperature. The strip was scanned using a ChemiDoc XRS + imaging system (Bio-Rad, USA). The scan signals were quantitatively analysed via ImageJ software (1.8.0). The GAPDH protein was used as a loading control.

### Immunofluorescence (IF)

(1) A cover slip was placed in a 6-well plate, the cell suspension was dropped on the cover slip, and cells were cultured for 4 h at 37  C in a 5% CO_2_ incubator. Cell medium was added to 2 ml, and the indicated drugs were added when the cell confluence rate was 50–60%. (2) The medium in the 6-well plate was discarded, the samples were rinsed 3 times with PBS, and 1 ml of 4% paraformaldehyde fixative was added to each well. The samples were fixed at room temperature for 15 min, after which the fixative was discarded and the samples were rinsed 3 times with PBS. (3) Blocking solution was added to the 6-well plate, which was subsequently blocked for 1 h at room temperature. (4) Primary antibodies (Proteintech, China) were added and the samples were incubated for 1 h at room temperature. (5) The membrane was rinsed with TBST three times for 10 min each. Fluorescent secondary antibodies (Beyotime) diluted in appropriate proportions were added, and the samples were incubated for 1 h at room temperature. (6) DAPI staining and sealing: the slides were rinsed 3 times with TBST for 10 min each time. DAPI working solution was added, and the samples were incubated at room temperature for 5 min, followed by three rinses with PBS for 5 min each. Two millilitres of antiquenching sealer was added to each well. (7) The samples were observed and photographed under a fluorescence microscope.

### Statistical analysis

All of the results are expressed as the means ± standard deviation. All data processing was performed using version 5.0 of the GraphPad Prism software. One-way ANOVA or t-test was used to compare the results between the treatments. At *p < 0.05, the results are considered to be significantly different.

## Results

### TEA alleviates HaCaT cell death induced by H_2_O_2_

By extraction and concentration, we obtained 80 ml of TEA concentrate, 105.3285 g, with a concentration of 1.3166 g/ml, which was stored at − 20 °C. The content of hypericin in TEA was 56.68 mg/ml according to HPLC.

**Fig. 1:**
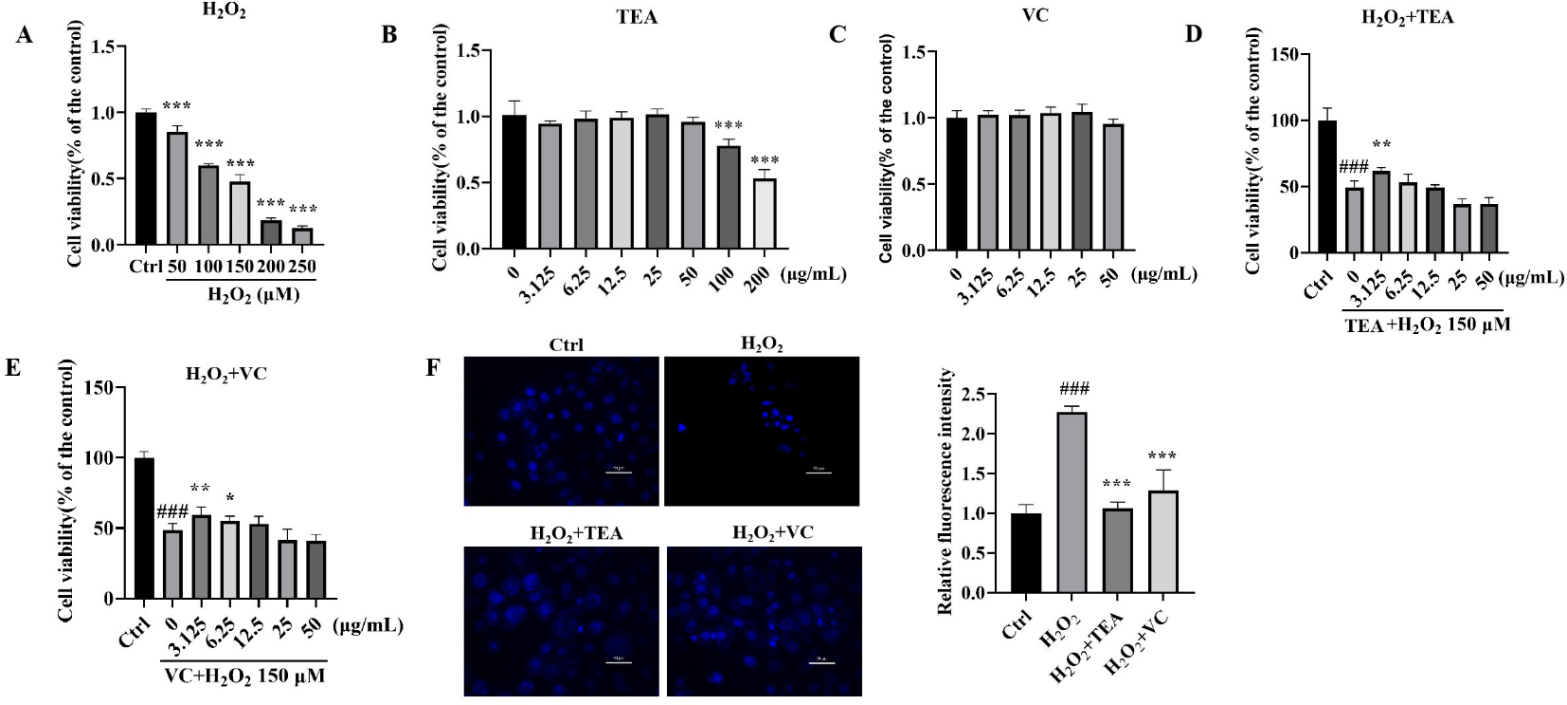
TEA alleviates HaCaT cell death induced by H_2_O_2_. (**A**) Effects of different concentrations of H_2_O_2_ on HaCaT cell activity. (**B**) Effects of different concentrations of TEA on HaCaT cell activity. (**C**) Effects of different concentrations of VC on HaCaT cell activity. (**D**) Effects of different concentrations of TEA on the activity of H_2_O_2_-treated HaCaT cells. (**E**) Effects of different concentrations of TEA on the activity of H_2_O_2_-treated HaCaT cells. (**F**) Hoechst 33,342 staining of cells. ^***^p < 0.001 versus Control; ^#^*p* < 0.05, ^##^*p* < 0.01 versus H_2_O_2_. (means ± SD, A-E, n = 6).

The MTT results (Fig. 1A–E), revealed that TEA had no adverse effect on cell activity concentrations ranging from 0 to 50 μM, and VC had no adverse effect on cell activity concentrations ranging from 0 to 50 μg/ml. For subsequent experiments, we selected H_2_O_2_ at 150 μM, TEA at 3.125 μg/mL, and VC at 3.125 μg/mL. The results of Hoechst 33,342 staining (Fig. 1F) reveald that the nuclei of the cells in the H_2_O_2_ group were more concentrated than those in the normal group were, the blue fluorescence was more obvious and the number of cells was significantly reduced. After treatment with TFA or VC, the concentration of solidified nuclei decreased, and the blue fluorescence decreased. These findings suggest that TEA and VC can alleviate H_2_O_2_-induced apoptosis.

### TEA ameliorates H_2_O_2_-induced oxidative damage in HaCaT cells

**Fig. 2:**
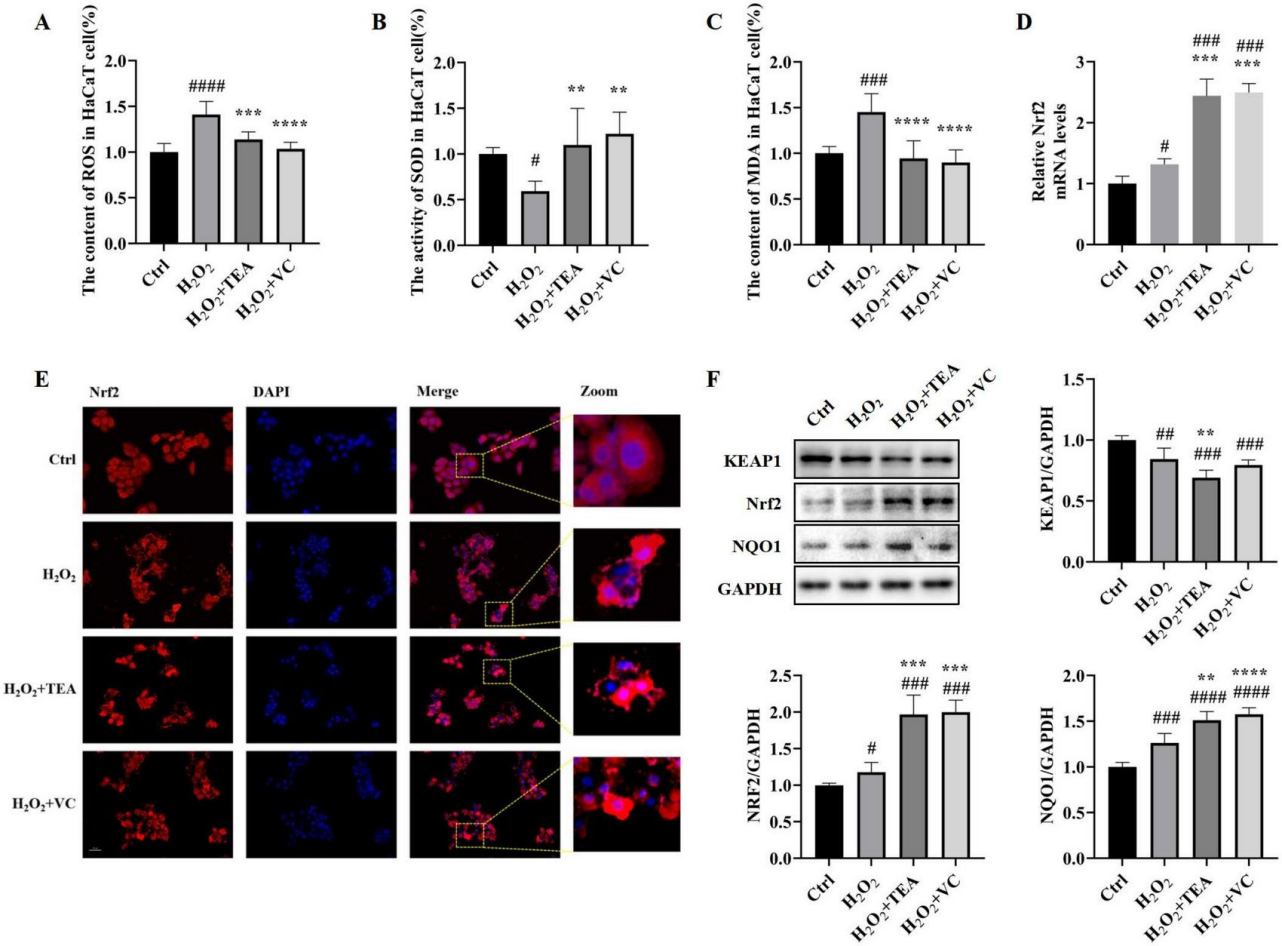
TEA ameliorates H_2_O_2_-induced oxidative damage in HaCaT cells. (**A**) ROS content in HaCaT cells. (**B**) SOD activity in HaCaT cells. (C) MDA content in HaCaT cells. (**D**) RT‒PCR detects Nrf2 mRNA levels in HaCaT cells. (**E**) IF detects Nrf2 protein expression in HaCaT cells. (**F**) WB was used to detect the protein expression levels of KEAP1, Nrf2 and NQO1 in HaCaT cells. ^#^*p* < 0.05, ^##^*p* < 0.01, ^###^*p* < 0.001 versus Control group; ^*^p < 0.05, **p < 0.01, ^***^p < 0.001 versus H_2_O_2_. (means ± SD, A-C, n = 6; D, F, n = 3).

Oxidative stress is caused by an imbalance between oxidants and antioxidants within cells^18^. The oxidative damage indices are shown in Fig. 2A–C. Compared with those in the control group, the ROS and MDA contents of HaCaT cells in the H_2_O_2_ group increased significantly, and the SOD activity decreased. Compared with those in the H_2_O_2_ group, the intracellular ROS and MDA contents in HaCaT cells decreased, and the SOD activity increased after treatment with TEA or VC. The PCR results indicated that the expression level of the Nrf2 gene slightly increased in the H_2_O_2_ group, and significantly increased in the H_2_O_2_ + TEA group and the H_2_O_2_ + VC group (Fig. 2D). WB and IF results revealed that the protein expression of Nrf2 in the H_2_O_2_ group was slightly increased, and the expression of Nrf2 protein in H_2_O_2_ + TEA group and H_2_O_2_ + VC group was significantly increased. IF (Fig. 2E was expressed in the cytoplasm or exhibited a dramatic nuclear shift. Nrf2 nuclear transfer was reflected in the fusion of red fluorescence representing Nrf2 and blue fluorescence representing nuclei in the H_2_O_2_, H_2_O_2_ + TEA and H_2_O_2_ + VC groups. The Western blotting results (Fig. 2F) revealed that the protein expression trend of KEAP1 was negatively correlated with that of Nrf2, and that the protein expression trends of NQO1 and KEAP1 were positively correlated with that of Nrf2. These results suggest that TEA protects cells from H_2_O_2_-induced oxidative damage, and that this protective effect operates through the KEAP1/Nrf2/NQO1 signalling pathway^19,20^. These results suggest that TEA protects cells from H_2_O_2_-induced oxidative damage, and that this protective effect operates through the KEAP1/Nrf2/NQO1 signalling pathway.

### TEA alleviates H_2_O_2_-induced mitochondrial oxidative damage and ameliorates mitochondrial biogenesis

**Fig. 3:**
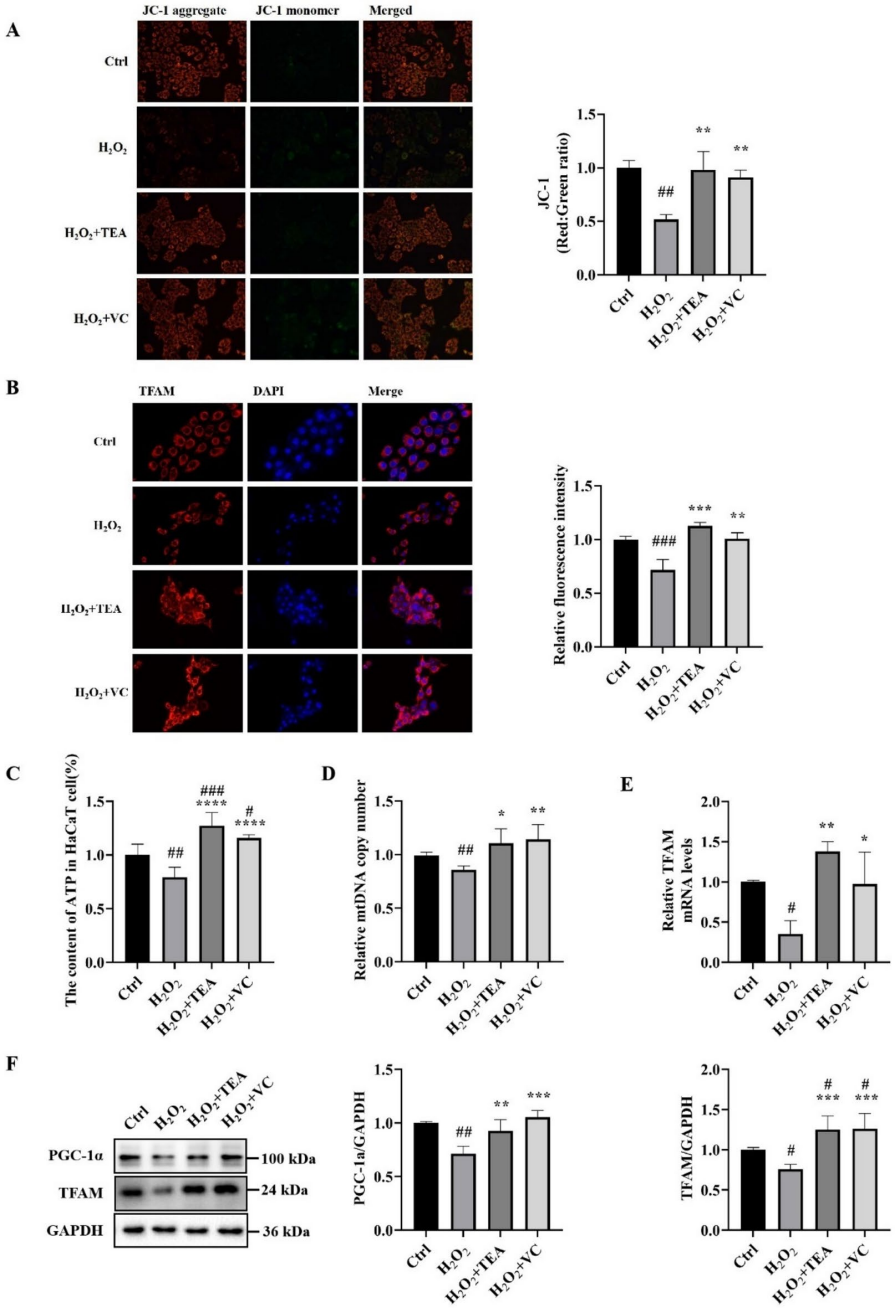

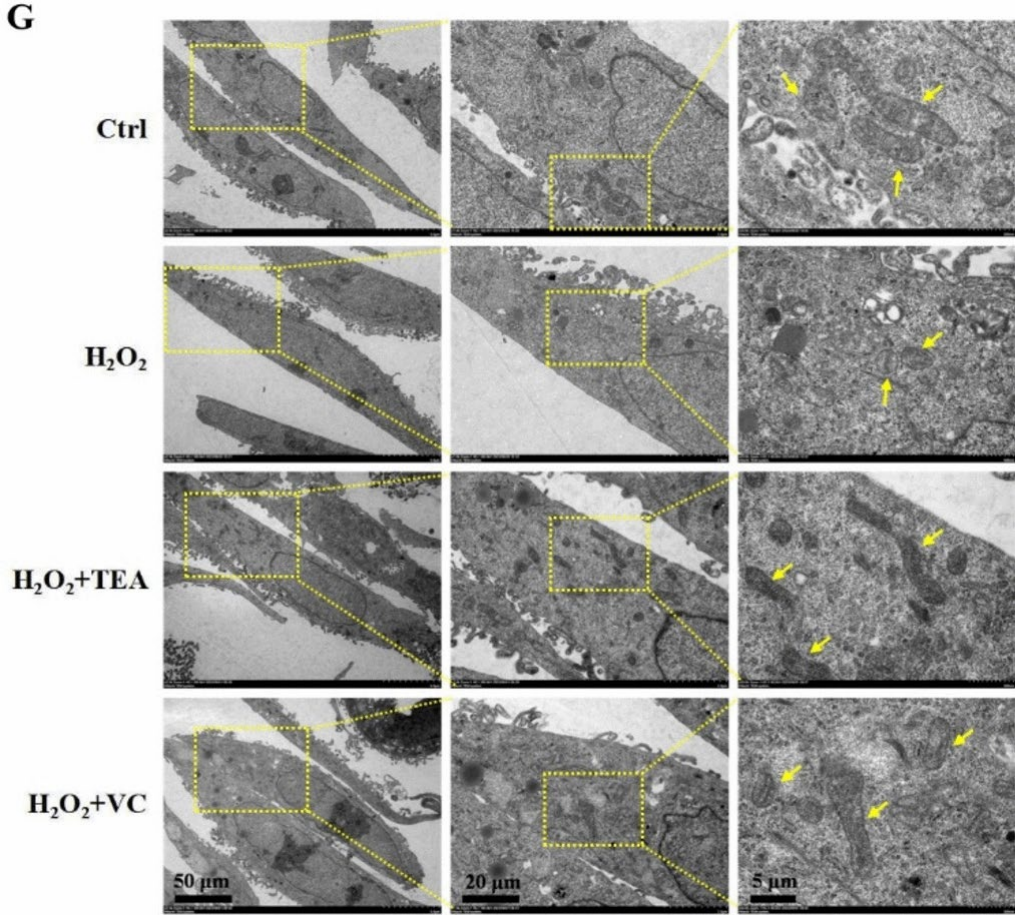
TEA alleviates H_2_O_2_-induced mitochondrial oxidative damage and ameliorates mitochondrial biogenesis. (**A**) Fluorescence microscopy detection of the mitochondrial membrane potential (JC-1). (**B**) IF detection of TFAM protein expression in HaCaT cells. (**C**) ATP content of HaCaT cells. (**D**) mtDNA copy number in HaCaT cells. (**E**) RT‒PCR detection of TFAM mRNA levels in HaCaT cells. (**F**) WB was used to detect the protein expression of KEAP1, Nrf2 and NQO1 in HaCaT cells. (**G**) Mitochondrial morphology of HaCaT cells by TEM. The arrows indicate mitochondria. ^#^*P* < 0.05, ^##^*P* < 0.01, ^###^*P* < 0.001 versus Control; ^*^P < 0.05, ^**^P < 0.01, ^***^P < 0.001, ^****^P < 0.00001 versus H_2_O_2_ (means ± SD, C-D, n = 6; E–F, n = 3).

The vast majority of ROS in cells are produced in mitochondria, which also makes mitochondria the first to be attacked by ROS under oxidative stress. The JC-1 results (Fig. 3A) revealed that, compared with that in the control group, the red fluorescence of cells in the H_2_O_2_ group was significantly weakened and the green fluorescence was slightly increased. Compared with that in the H_2_O_2_ group, the red fluorescence of cells in the H_2_O_2_ + TEA group and the H_2_O_2_ + VC group was obvious, and the green fluorescence was weak. These findings indicate that TEA and VC are able to prevent H_2_O_2_ destruction to maintain the normal mitochondrial membrane potential. H_2_O_2_ reduced the intracellular ATP content (Fig. 3C) and mtDNA copy number (Fig. 3D), and the intracellular ATP content and mtDNA copy number increased significantly after TEA and VC treatment.

PGC-1α is a transcriptional activator of mitochondria-associated genes such as TFAM, which is an important factor involved in mtDNA transcriptional activation and the regulation of mtDNA copy number^21^. The RT-PCR results (Fig. 3E) revealed that H_2_O_2_ reduced TFAM mRNA levels and that TFAM mRNA levels increased significantly after TEA and VC treatment. WB results (Fig. 3F) revealed that H_2_O_2_ decreased PGC-1α and TFAM protein expression, and that PGC-1α and TFAM protein expression increased significantly after TEA and VC treatment. The IF results of TFAM (Fig. 3B) were consistent with the WB results. By observing the mitochondrial morphology of the cells via transmission electron microscopy (Fig. 3G), we found that compared with those in the control group, most of the mitochondria in the H_2_O_2_ group were swollen and rounded, and fewer mitochondrial crest cells were observed. Compared with those in the H_2_O_2_ group, the cells in the H_2_O_2_ + TEA group and the H_2_O_2_ + VC group also had swollen and rounded mitochondria, but more normal mitochondria.

The above results show that H_2_O_2_ treatment changes the morphology of cell mitochondria and impairs their function. TEA can protect HaCaT cells from H_2_O_2_-induced damage to mitochondrial morphology and function, protect against mitochondrial biogenesis, and effectively protect against mitochondrial oxidative stress.

### TEA alleviates H_2_O_2_-induced decreases in the expression of mtDNA-encoding genes and proteins

**Fig. 4:**
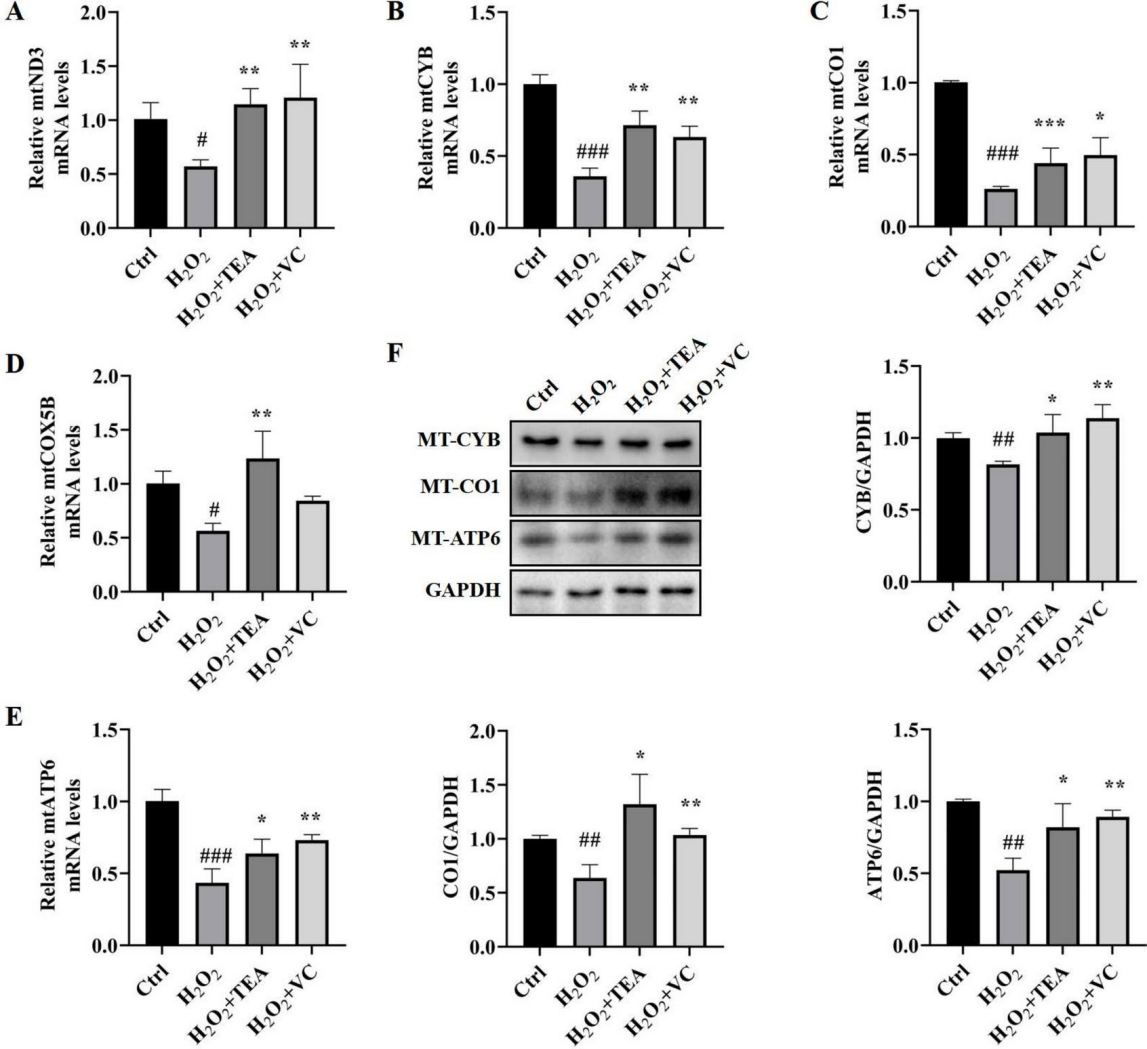
TEA alleviates H2O2-induced decreases in the expression of mtDNA-coding genes and proteins. (**A**) Relative mtND3 mRNA levels in HaCaT cells. (**B**) Relative mtCYB mRNA levels in HaCaT cells. (**C**) Relative mtCO1 mRNA levels in HaCaT cells. (**D**) Relative mtCOX5B mRNA levels in HaCaT cells. (**E**) Relative mtATP6 mRNA levels in HaCaT cells. (**F**) WB detection of the protein expression levels of MT-CYB, MT-CO1 and MT-ATP6 in HaCaT cells. ^#^*P* < 0.05, ^##^*P* < 0.01, ^###^*P* < 0.001 versus Control, ^*^P < 0.05, ^**^P < 0.01, ^***^P < 0.001 versus H_2_O_2_ (means ± SD, n = 3).

In the previous experiments, we showed that H_2_O_2_ reduces the mtDNA copy number, and that TEA and VC can reverse the effect of H_2_O_2_. On this basis, we examined the expression of mRNAs and proteins in the mtDNA-coding genes (Fig. 4). Compared with those in the control group, the levels of mtND3, mtCYB, mtCO1, mtCOX5B, and mtATP6 mRNAs in cells in the H_2_O_2_ group were significantly llower, and the levels of these mRNAs were restored after treatment with TEA and VC. The WB results were consistent with the RT‒PCR results, and the expression of the MT-CYB, MT-CO1, and MT-ATP6 proteins in H_2_O_2_ treated cells was significantly lower than that in the control cells, and the levels of these proteins were restored after treatment with TEA and VC.

### TEA does not protect against H_2_O_2_-induced oxidative stress in HaCaT cells after Nrf2 inhibition

**Fig. 5:**
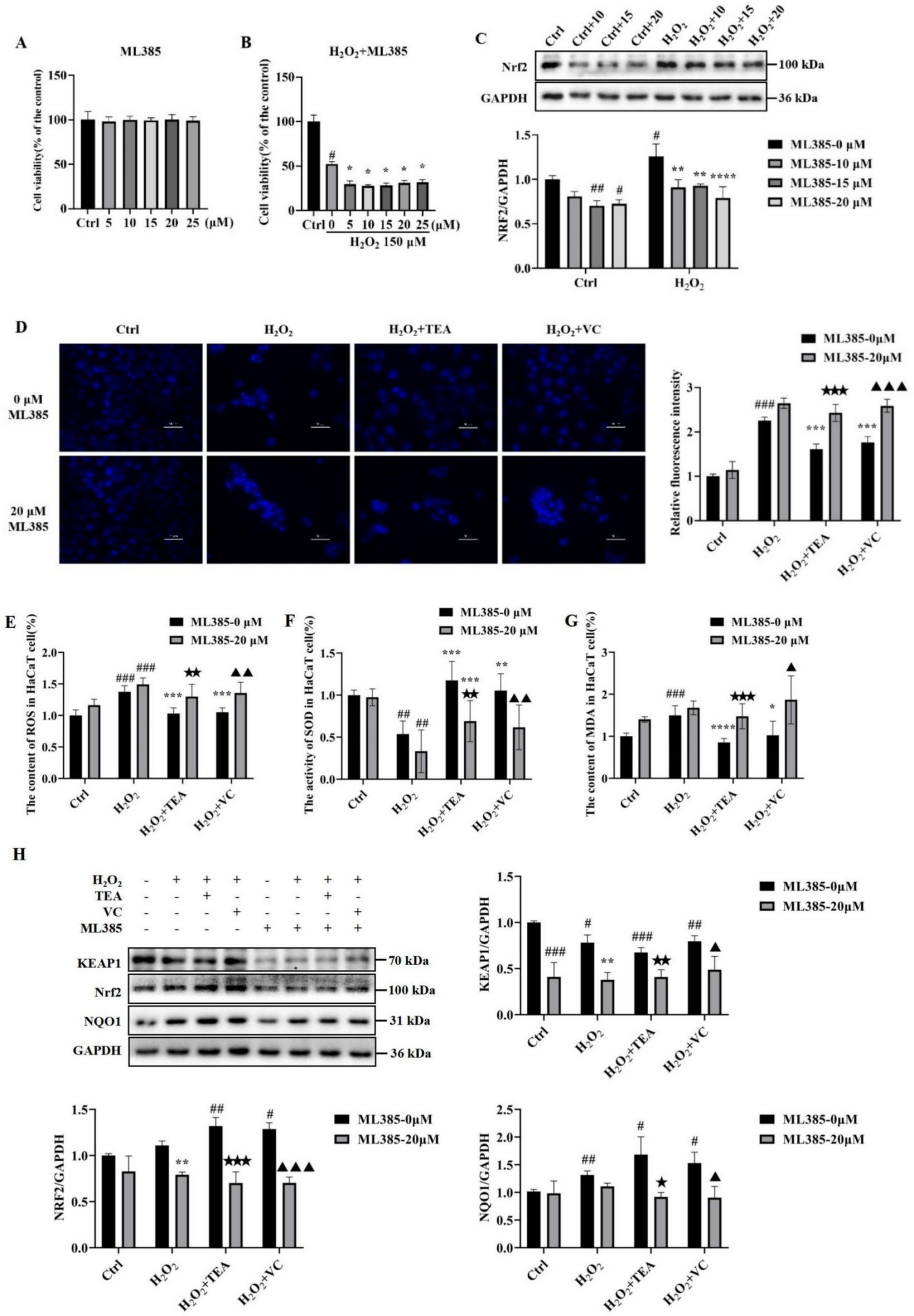
TEA ameliorates H_2_O_2_-induced oxidative damage in HaCaT cells through the KEAP1/Nrf2/NQO1 signalling pathway. (**A**) Effects of different concentrations of ML385 on HaCaT cell activity. (**B**) Effects of different concentrations of ML385 on the activity of H_2_O_2_-treated HaCaT cells. ^*^P < 0.05 versus Control, ^#^*P* < 0.05 versus H_2_O_2_ (means ± SD, n = 6). (**C**) WB analysis of the effects of different concentrations of ML385 on Nrf2 protein expression in HaCaT cells. ^#^*P* < 0.05, ^##^*P* < 0.01 versus Control, ^**^P < 0.01, ^****^P < 0.0001 versus H_2_O_2_ (means ± SD, n = 3). (**D**) Hoechst 33,342 staining of HaCaT cells. (**E**) Effect of ML385 (20 μM) on the ROS content in HaCaT cells. (**F**) Effect of ML385 (20 μM) on the SOD activity in HaCaT cells. (**G**) Effects of ML385 (20 μM) on the MDA content in HaCaT cells. (**H**) WB was used to detect the effects of ML385 (20 μM) on the protein expression levels of KEAP1, Nrf2, and NQO1 in HaCaT cells. ^#^*P* < 0.05, ^##^*P* < 0.01, ^###^*P* < 0.001 versus Control; ^*^P < 0.05, ^**^P < 0.01, ^***^P < 0.001 versus H_2_O_2_; ^★^*P* < 0.05, ^★★^*P* < 0.01,^★★★^*P* < 0.001 versus H_2_O_2_ + TEA; ^▲^*P* < 0.05, ^▲▲^*P* < 0.01,^▲▲▲^*P* < 0.001 versus H_2_O_2_ + VC (means ± SD, E‒G, n = 6; H, n = 3).

To further explore whether the KEAP1/Nrf2/NQO1 signalling pathway plays a major role in the effects of TEA, we used a Nrf2 inhibitor ML385 (20 μM) (Fig. 5A–C) as a tool for follow-up studies.

MTT assays revealed that ML385 had no effect on the activity of HaCaT cells at concentrations ranging from 5 to 25 μM (Fig. 5A). The application of ML385 o in addition to H_2_O_2_ further reduced cell viability (Fig. 5B). Since the inhibitory effect of 5–25 μM ML385 on cells treated with H_2_O_2_ was almost uniform (Fig. 3B), we further screened the optimal dose of ML385 via WB. The WB results (Fig. 5C) revealed that the inhibition of Nrf2 was most pronounced when the ML385 concentration was 20 μM, so we chose 20 μM as the working concentration of ML385.

After combination with ML385, the nuclei of the H_2_O_2_ + TEA group and H_2_O_2_ + VC group stained with Hoechst 33,342 were significantly concentrated, the blue fluorescence was significantly increased, the number of cells decreased, apoptosis was evident (Fig. 5D), and TEA and VC could no longer reverse H_2_O_2_-induced apoptosis. After combination with ML385, the ROS and MDA contents (Fig. 5E,G) of the H_2_O_2_ + TEA group and the H_2_O_2_ + VC group increased significantly, the SOD activity (Fig. 5F) decreased significantly, and TEA and VC could no longer reverse the H_2_O_2_-induced oxidative damage. After combination with ML385, the protein expression of KEAP1, Nrf2 and NQO1 in the H_2_O_2_ + TEA group and the H_2_O_2_ + VC group was inhibited (Fig. 5H), and TEA and VC no longer promoted the protein expression of Nrf2 and NQO1 proteins. These results show that the antioxidant effect of TEA is indeed exerted through the KEAP1/Nrf2/NQO1 signalling pathway.

### TEA loses its protective effect against H_2_O_2_-induced mitochondrial damage in HaCaT cells after Nrf2 inhibition

**Fig. 6:**
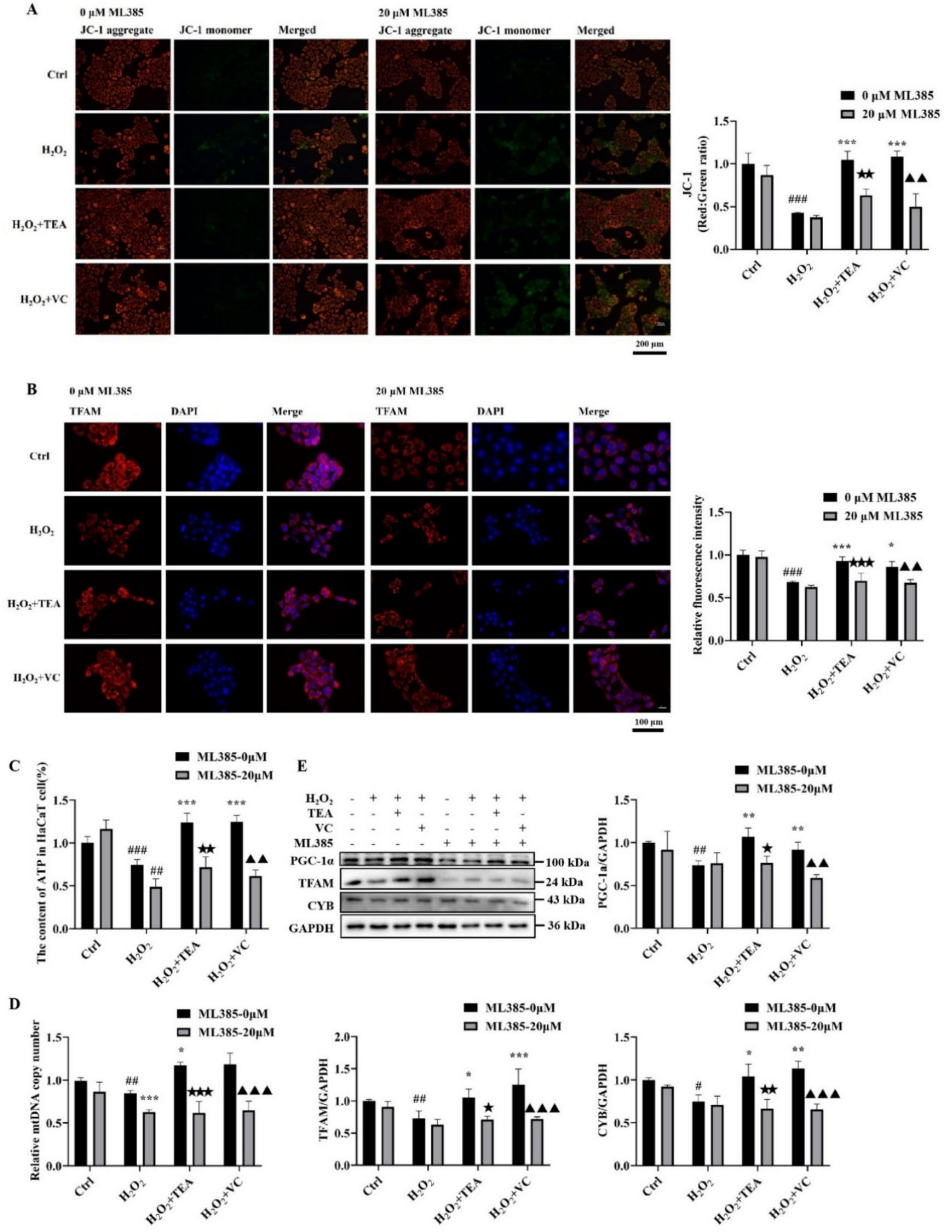
TEA alleviates H2O2-induced mitochondrial oxidative damage and improves mitochondrial biogenesis through the Nrf2/TFAM/mtDNA/ETC pathway. (**A**) Fluorescence microscopy was used to detect the effect of ML385 on the mitochondrial membrane potential in HaCaT cells. (**B**) IF was used to measure the effect of ML385 on TFAM protein in HaCaT cells. (**C**) Effect of ML385 on the ATP content in HaCaT cells. (**D**) Effect of ML385 on mtDNA copy number in HaCaT cells. (**E**) Effects of ML385 on PGC-1α, TFAM and MT-CYB protein expression levels in HaCaT cells. ^#^*P* < 0.05, ^##^*P* < 0.01, ^###^*P* < 0.001 versus Control; ^*^P < 0.05, ^**^P < 0.01, ^***^P < 0.001 versus H_2_O_2_; ^★^*P* < 0.05, ^★★^*P* < 0.01,^★★★^*P* < 0.001 versus H_2_O_2_ + TEA; ^▲^*P* < 0.05, ^▲▲^*P* < 0.01,^▲▲▲^*P* < 0.001 versus H_2_O_2_ + VC (means ± SD, C-D, n = 6; E, n = 3).

Furthermore, to explore whether Nrf2-mediated antioxidant pathways have a protective effect against mitochondrial damage, we tested mitochondrial indices via the application of ML385. JC-1 staining (Fig. 6A) revealed that H_2_O_2_-induced changes in the mitochondrial membrane potential were more pronounced after combination with ML385, with red fluorescence weakening and green fluorescence increasing. Significant green fluorescence could still be observed after TEA and VC treatment, indicating that TEA and VC treatment could no longer reverse H_2_O_2_-induced changes in the mitochondrial membrane potential after Nrf2 inhibition.

Compared with those in the 0-ML385 group, the ATP content (Fig. 6C) and mtDNA copy number (Fig. 6D) in the H_2_O_2_, H_2_O_2_ + TEA and H_2_O_2_ + VC groups were significantly lower. When Nrf2 is inhibited, TEA and VC are not able to reverse H_2_O_2_-induced reductions in ATP content and mtDNA copy number. WB results (Fig. 6E) showed that after combined use with ML385, the H_2_O_2_ + TEA group and H_2_O_2_ + VC groups had a statistically significant decrease in the expression of the proteins PGC-1α and TFAM associated with mitochondrial biogenesis and the mtDNA-encoded protein CYB. The WB results of TFAM were consistent with the IF results (Fig. 6B).

Nrf2 is closely related to mitochondrial function, and when Nrf2 is inhibited, TEA no longer has a protective effect on mitochondria, and PGC-1α and TFAM may be downstream targets for Nrf2. These results further confirmed that TEA alleviates H_2_O_2_-induced mitochondrial dysfunction in HaCaT cells through Nrf2 signalling.

### TEA does not have a protective effect against H_2_O_2_-induced mitochondrial damage in HaCaT cells after TFAM knockdown

**Fig. 7:**
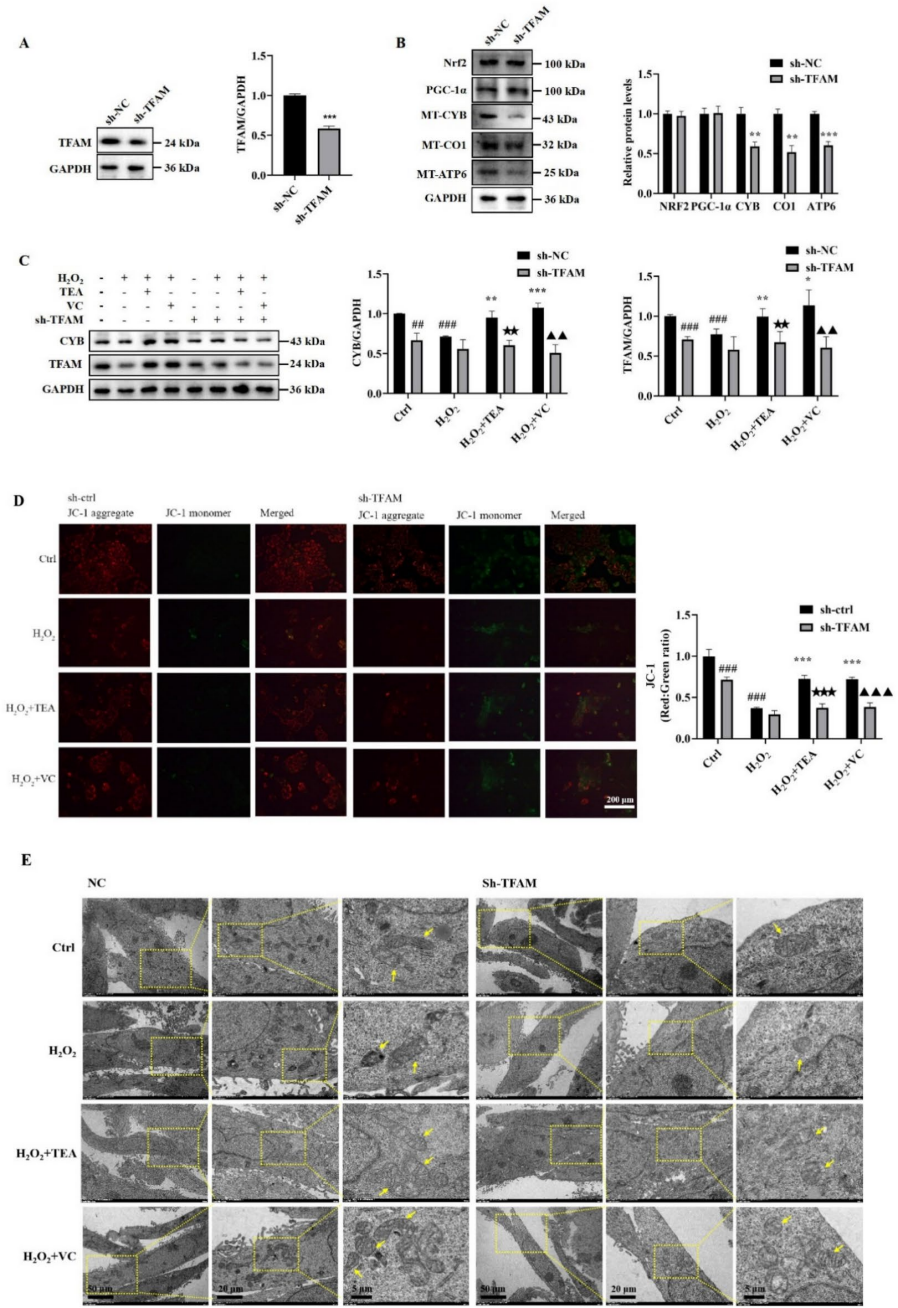
Effects of TFAM knockdown on mitochondrial function in HaCaT cells. (**A**) Effect of TFAM knockdown on TFAM protein expression in HaCaT cells. (**B**) Effects of TFAM knockdown on the protein expression of Nrf2, PGC-1α, MT-CYB, MT-CO1 and MT-ATP6 in HaCaT cells. (**C**) The protein expression of MT-CYB and TFAM after the indicated treatment. (**D**) The effect of TFAM knockdown on the mitochondrial membrane potential of HaCaT cells was detected via fluorescence microscopy. (**E**) The effect of TFAM knockdown on the mitochondrial morphology of HaCaT cells was observed via transmission electron microscopy. The arrows indicate mitochondria. A-B: ^**^P < 0.01, ^***^P < 0.001 versus sh-NC; C: ^##^*P* < 0.01, ^###^*P* < 0.001 versus Control; ^*^P < 0.05, ^**^P < 0.01, ^***^P < 0.001 versus H_2_O_2_;^★★^*P* < 0.01 versus H_2_O_2_ + TEA; ^▲▲^*P* < 0.01 versus H_2_O_2_ + VC (means ± D, n = 3).

Finally, to explore the relationship between TFAM and Nrf2 and the effect of TFAM on mitochondrial morphological function, lentiviral infection was used to construct a stable HaCaT cell line with low expression of the TFAM gene. The TFAM knockdown efficiency was verified by WB to be approximately 50% (Fig. 7A). In addition, after TFAM knockdown, there was no significant change in the protein expression of Nrf2 or PGC-1α, suggesting that TFAM is located downstream of Nrf2 and PGC-1α and that the regulation of TFAM by Nrf2 is unidirectional. The decreased expression of the MT-CYB, MT-CO1 and MT-ATP6 proteins was significant, suggesting that TFAM has a regulatory effect on mtDNA-coded proteins, and that TFAM is essential for maintaining the normal function of the mitochondrial electron respiratory chain (Fig. 7B). When TFAM was knocked down, TEA and VC no longer alleviated the H_2_O_2_-induced decrease in TFAM and MT-CYB protein expression (Fig. 7C), and no longer protected the mitochondria to maintain normal morphology and mitochondrial membrane potential (Fig. 7D,E). These results suggest that TEA plays a mitochondrial protective role by promoting the expression of TFAM, which is the downstream target protein of TEA.

## Discussion

In this study, H_2_O_2_ treatment caused cellular oxidative stress and apoptosis, including decreased cell activity, nuclear contraction, increased levels of the oxidative stress products ROS and MDA, and decreased activity of the antioxidant enzyme SOD, which indicated that the skin oxidative stress model was successfully constructed.

Hyperin is one of the main components of *AM*. Hyperin has been reported to protect rat ovarian cells from H_2_O_2_-induced oxidative stress. In this study, H_2_O_2_ caused an increase in the ROS and MDA contents and a decrease in the activity of SOD in HaCaT cells. TEA treatment alleviated the above phenomena caused by H_2_O_2_. Nrf2 is considered one of the most sensitive factors to oxidative stress^22,23^. In this study, Nrf2 was shown to play a dominant role during OS in HaCaT cells. More importantly, TEA therapy can stimulate Nrf2 signalling molecules and initiate downstream antioxidant responses. Therefore, this study aimed to explore the molecular mechanism of the antioxidant action of TEA and the downstream target proteins of Nrf2-mediated signalling pathway.

At present, researchers generally believe that under normal circumstances, Nrf2 is localized in the cytoplasm expressed at low levels in the body, and forms a ternary complex with KEAP1, which is degraded by KEAP1 ubiquitination^24^. Redox-sensitive cysteine residues in KEAP151 (e.g., Cys273, Cys288, and Cys1) are modified when the body is subjected to oxidative stress or the presence of electrophilic compounds, resulting in Nrf2 dissociation and subsequent translocation into the nucleus. In the nucleus, Nrf2 binds to small Maf (sMaf) proteins and other transcription factors such as SP-1 or the c-JUN heterodimer and binds to specific sequences of ARE, inducing its transactivation, and activating downstream antioxidant enzymes, such as NAD(P)H:quinone oxidoreductase 1 (NQO1) and haem oxygenase-1 (HO-1).

WB results revealed that H_2_O_2_ led to a decrease in the protein expression of KEAP and an increase in the protein expression of Nrf2 and NQO1. Through immunofluorescence, we found that when oxidative stress occurs in cells, nuclear displacement of the Nrf2 protein occues. These results are consistent with the theory. After TEA treatment, the protein expression of KEAP1 in cells was further reduced, the expression of Nrf2 and NQO1 proteins was further increased, and TEA treatment promoted the nuclear displacement of the Nrf2 protein. These findings indicate that TEA plays an antioxidant role by activating the Nrf2 signalling pathway.

We found that during oxidative stress, the mitochondrial membrane potential decreases, the ATP content decreases, mitochondrial swelling and deformation occur, mitochondrial crest breaks, and the mitochondrial membranes are distrupted. After TEA treatment, the above phenomenon was significantly reversed. These results suggest that H_2_O_2_-induced impairment of mitochondrial morphological function can be reversed by TEA. Mitochondria are downstream target organelles of Nrf2.

Mitochondria are semiautonomous organelles with unique DNA and genetic codes that play important roles in amino acid synthesis, lipid metabolism, energy production and other metabolic processes^25^. However, MtDNA is susceptible to mutation because of a lack of histone protection and the high risk of oxygen radical attack. Therefore, mtDNA mutations are ten or even one hundred times more common than they are in the nuclear genome^26^. At present, Nrf2 has two main regulatory effects on mitochondria: Nrf2 mediates mitochondrial autophagy through P62 and mitochondrial biogenesis is mediated through PGC-1α^27^. TFAM is an essential mtDNA packaging protein required for mtDNA replication and transcription. Under pathological conditions, disruption of TFAM can lead to mtDNA depletion and insufficient mitochondrial bioenergetics. We found that during OS, the mtDNA copy number decreased, PGC-1α and TFAM protein expression was inhibited, and mRNA and protein expression of mitochondrial ETC genes encoded by mtDNA was inhibited. After TEA treatment, the above phenomenon was significantly revers. These results suggest that H_2_O_2_-induced mitochondrial damage is associated with the inhibition of mitochondrial biogenesis and thus mitochondrial ETC function, and that this inhibition can be reversed by TEA.

To further explore whether Nrf2 is directly related to mitochondria, especially TFAM, we introduced the Nrf2 inhibitor ML385 for follow-up experiments. ML385 acts on the NEH1 domain of Nrf2 and inhibits Nrf2 binding to the ARE, thereby inhibiting the transcriptional activity of Nrf2. We found that the ability of TEA to alleviate H_2_O_2_-induced apoptosis and oxidative stress was inhibited by ML385. Moreover, the protein expression of KEAP1, Nrf2, and NQO1 was also inhibited. These results further confirmed that TEA exerts antioxidant effects through the Nrf2 pathway. The mitochondrial indices revealed that TEA did not increase ATP content or mtDNA copy number, or PGC-1α, TFAM or MT-CYB protein expression after the application of ML385. These findings suggest that Nrf2 is closely related to mitochondrial function and mitochondrial biogenesis.

In addition, we constructed a TFAM-stable low-expression HaCaT cell line. The WB results revealed that the protein expression of Nrf2 and PGC-1 did not change significantly when TFAM was knocked down, and that the protein expression of MTCYB, MTCO1 and MTATP6 decreased significantly. These findings indicate that TFAM is a downstream protein of Nrf2 and PGC-1α, and that TFAM plays a key role in the expression of mtDNA-coded proteins. Therefore, we explored the nucleus-to-mitochondrion communication pathway: Nrf2/PGC-1α/TFAM/mtDNA/ETC.

## Conclusion

This study confirmed the role of Nrf2 in H_2_O_2_-induced oxidative damage in HaCaT cells, and explored the association between Nrf2 and TFAM. This study is the first to demonstrate that TEA protects HaCaT cells from H_2_O_2_-induced oxidative damage and mitochondrial damage through the KEAP1/Nrf2/NQO1/PGC-1α/TFAM pathway.

## Supplementary Information

Supplementary Information.

## Data Availability

Data is provided within the manuscript or supplementary information files.

## Abbreviations

ETC: Electron transfer chain
H_2_O_2_: Hydrogen peroxide
HO-1: Haem oxygenase 1
KEAP1: Kelch-like ECH-associated protein 1
LSP: Lipopolysaccharides
MDA: Malondialdehyde
MT-ATP6: Mitochondrial adenosine triphosphate 6
MT-CO1: Mitochondrial cytochrome C oxidase 1
MT-COX5B: Mitochondrial cytochrome C oxidase subunit 5B
MT-CYB: Mitochondrial cytochrome B
MtDNA: Mitochondrial DNA
MT-ND1: Mitochondrial NADH dehydrogenase 1
NADPH: Nicotinamide adenine dinucleotide phosphate
Neh: Nrf2-ECH homology domains
NQO1: NADH Quinone Oxidoreductase 1
Nrf2: Nuclear factor-E2-related factor
OS: Oxidative stress
PGC-1α: Peroxisome proliferator–activated receptor gamma coactivator-1 alpha
ROS: Reactive oxygen species
SOD: Superoxide Dismutase
TFAM: Mitochondrial transcription factor A
UVR: Ultraviolet radiation
VC: Vitamin C
