# Supplementary material for: The total extract of Abelmoschus manihot (L.) medic flowers (TEA) mediated Nrf2-TFAM signalling to regulate mitochondrial antioxidant mechanism
Source: Sci Rep. 2025 Jan 10;15:1614. doi: 10.1038/s41598-024-84022-x (PMC11723989; doi:10.1038/s41598-024-84022-x)
Supplement: Supplementary file 1 — Supplementary Information. [file 41598_2024_84022_MOESM1_ESM.pdf]

Analysis report on the concentration of hypericin in the flowers of *Abelmoschus manihot* (L.) Medic UPLC-Q/TOF-MS

1. Materials and methods

1.1. Equipment and materials

SCIEX X-500R four-pole time-of-flight mass spectrometer (AB SCIEX, America); TurboIonSpray ion source (AB SCIEX, America); Waters ACQUITY I-Class Plus UPLC (Waters, America); Thermo ST40R Heraeus sepatech (Thermo, America); IKA Mini Vortex Mixer (KA, German); AUW220D Electric balance (Shimadzu, Japan).

Methanol, acetonitrile and formic acid (Merck, German), Milli-Q ultrapure water (Millipore, America). All other reagents were analytically pure.

1.2. Solution preparation

1.2.1 Test solution

Precision absorption of 40~50 mg/ml of *Abelmoschus manihot* (L.) Medic flower sample solution 20 μl, add methanol 980 μl dilution 50 times, vortex mix; Accurately absorb and dilute 50 times the sample solution of 25 μl, add methanol 975 μl to dilute 40 times, vortex mix; The above diluted sample solution of 100 μl was precisely absorbed, 900 μl methanol was added, and then mixed with vortex to obtain the *Abelmoschus manihot* (L.) Medic flower sample solution diluted 20,000 times with a crude concentration of 2~2.5 μg/ml. After centrifugation at 12000 rpm/min for 20 min, the supernatant was taken and injected.

1.2.2 Control solution

Accurately weigh 1 mg of hypericin reference substance in a centrifuge tube, add 1 ml of methanol and vortex to mix well to obtain a 1 mg/ml concentration solution. Pipette 5 μl of 1 mg/ml solution and add 950 μl of methanol to a new centrifuge tube, vortex and mix well to obtain a 50 μg/ml concentration solution. Pipette 100 μl of 50 μg/ml solution into a new centrifuge tube, and add 900 μl of methanol to obtain a 5 μg/ml concentration solution; Pipette 800 μl of 5 μg/ml solution into a new centrifuge tube, add 200 μl of methanol to obtain a 4 μg/ml concentration solution, and perform the same operation to obtain a control solution of 2 μg/ml, 1 μg/ml, 0.1 μg/ml and 0.01 μg/ml concentration. All control solutions were centrifuged at 12000 rpm/min for 20 min, supernatants were taken, and samples were injected.

1.3 Chromatographic and mass spectrometry conditions

Chromatographic conditions: ACQUITY UPLC BEH C18 (100 mm× 2.1 mm, 1.7 μm) Column, mobile phase 0.1% acetonitrile formate (A)-0.1% formic acid water (B), gradient elution procedure: 0～1 min, 98%~98%B; 1~5 min, 98%~80%B; 5~8 min, 80%~80%B; 8~13 min,

80%~50%B; 13~16 min, 50%~10%B; 16~19 min, 10%~2%B；19~19.1 min, 2%~98%B; 19.1~22 min, 98%~98%B. Velocity：0.3 mL/min; Sample tray temperature: 8 °C; Column temperature: 40 °C; Injection volume: 2 µl.

Mass spectrometry conditions: Detection was performed in electrospray ionization (ESI-) negative ion mode. The ESI source conditions after chromatographic separation are as follows: Ion Source Gas1 (Gas1): 55, Ion Source Gas2 (Gas2): 55, Curtain gas (CUR): 35, source temperature: 600°C; Ion Sapary Voltage Floating (ISVF)-4500 V; TOF MS scan m/z range: 50-1500D, TOF MS scan accumulation time 0.25s/spectra; Secondary mass spectrometry was obtained by MRM HR, and the MRM parameters are shown in Table 1.

Table S1 Hyperoside MRM parameters

Compound ID	Precursor ion (Da)	Fragment ion (Da)	Accumulation time (sec)	Declustering potential (V)	Collision energy (V)	Retention time (min)	Adduct
JSTG	463.09	300.0279	0.1	-80	-35	6.15	[M-H]

2. Results & Analysis

2.1 MRM spectra of *Abelmoschus manihot* (L.) Medic flower test and hypericin reference substance

The "1.2.1Test solution" and "1.2.2 Control solution" were analyzed by UHPLC-Q/TOF-MS system, and the MRM patterns of the test product and hyperoside control were obtained (Fig. 1).

A

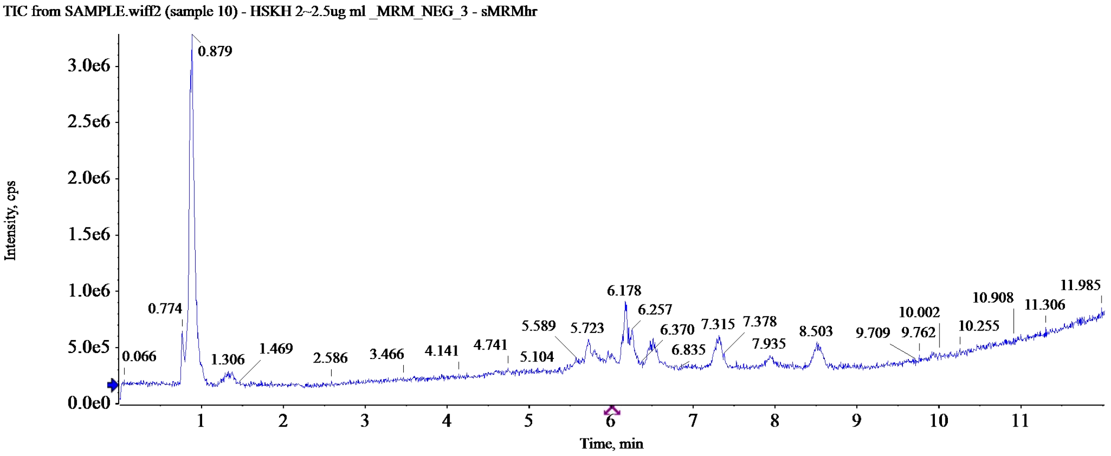

B

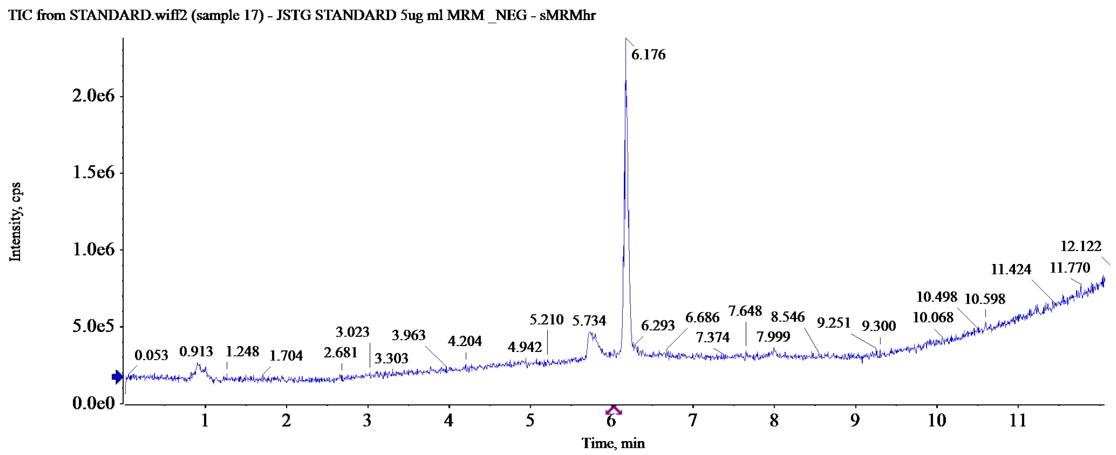

Figure S1 UHPLC-Q/TOF-MS MRM spectrum of *Abelmoschus manihot* (L.) Medic flower (negative ion mode);

A-*Abelmoschus manihot* (L.) Medic flower test solution; B-hyperoside control solution

2.2 Linear relationship investigation

The diluted samples of each concentration of reference substance and *Abelmoschus manihot* (L.) Medic flowers were analyzed byUPLC-Q/TOF-MS system, and the peak area was recorded, and the results are shown in Table 2. The linear regression equation for hyperoside was obtained by fitting the control concentration and peak area (Figure 3), i.e., $y = 239104x + 1379.4$ and $r = 0.9999$ (where x is the solution concentration and y is the peak area).

Table S2 Peak area results of reference products at each concentration of hypericin

Sample Name	Actual Concentration （ug/ml）	Formula	Retention Time	Area
JSTG STANDARD 0.01ug ml MRM _NEG	0.01	C21H20O12	6.19	4.01E+03
JSTG STANDARD 0.1ug ml MRM _NEG	0.1	C21H20O12	6.18	2.87E+04
JSTG STANDARD 1ug ml MRM _NEG	1	C21H20O12	6.18	2.47E+05
JSTG STANDARD 2ug ml MRM _NEG	2	C21H20O12	6.17	4.66E+05
JSTG STANDARD 4ug ml MRM _NEG	4	C21H20O12	6.18	9.53E+05
JSTG STANDARD 5ug ml MRM _NEG	5	C21H20O12	6.18	1.21E+06

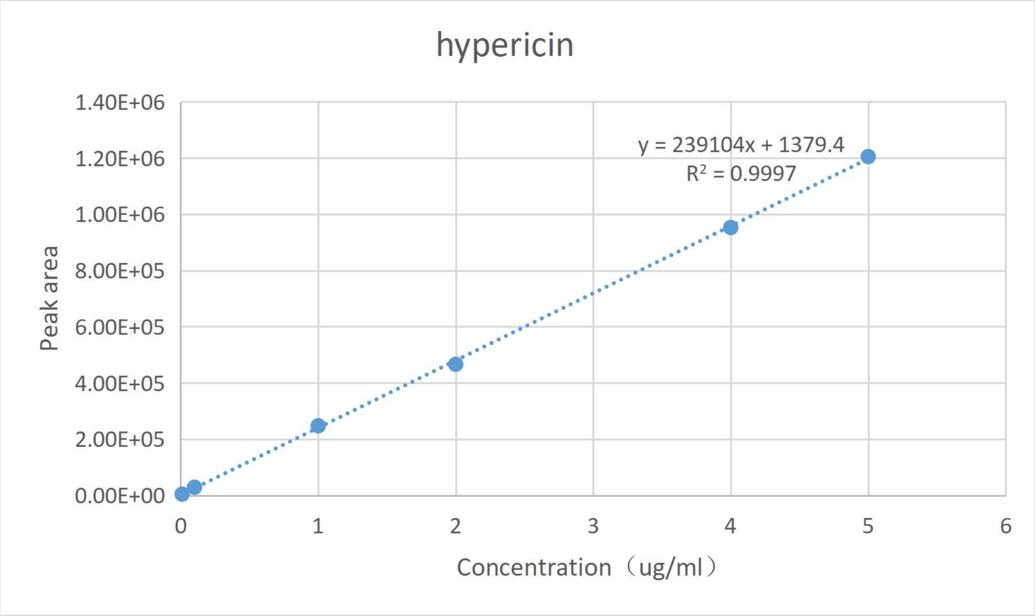

Figure S2 Linear regression equation for hypericin reference substance

2.3 Sample content determination

"1.2.1 Test solution" was analyzed by UPLC-Q/TOF-MS system, three needles were injected consecutively, the peak area of the sample was recorded, and the peak area was substituted into the standard curve y = 239104x + 1379.4 to obtain the diluted sample concentration (Table 3). The average concentration of the three needles was 2.834 µg/ml, which was multiplied by the dilution factor of 20,000 to calculate the original concentration of 56.68 mg/ml.

Table S3 Calculation results of dilution peak area and concentration of *Abelmoschus manihot* (L.) Medic flower samples

Sample Name	Retention Time	Area	Concentration（µg/ml）
HSKH 2~2.5ug ml _MRM_NEG_1	6.18	6.67E+05	2.783812
HSKH 2~2.5ug ml _MRM_NEG_2	6.21	7.22E+05	3.014256
HSKH 2~2.5ug ml _MRM_NEG_3	6.18	6.48E+05	2.703512

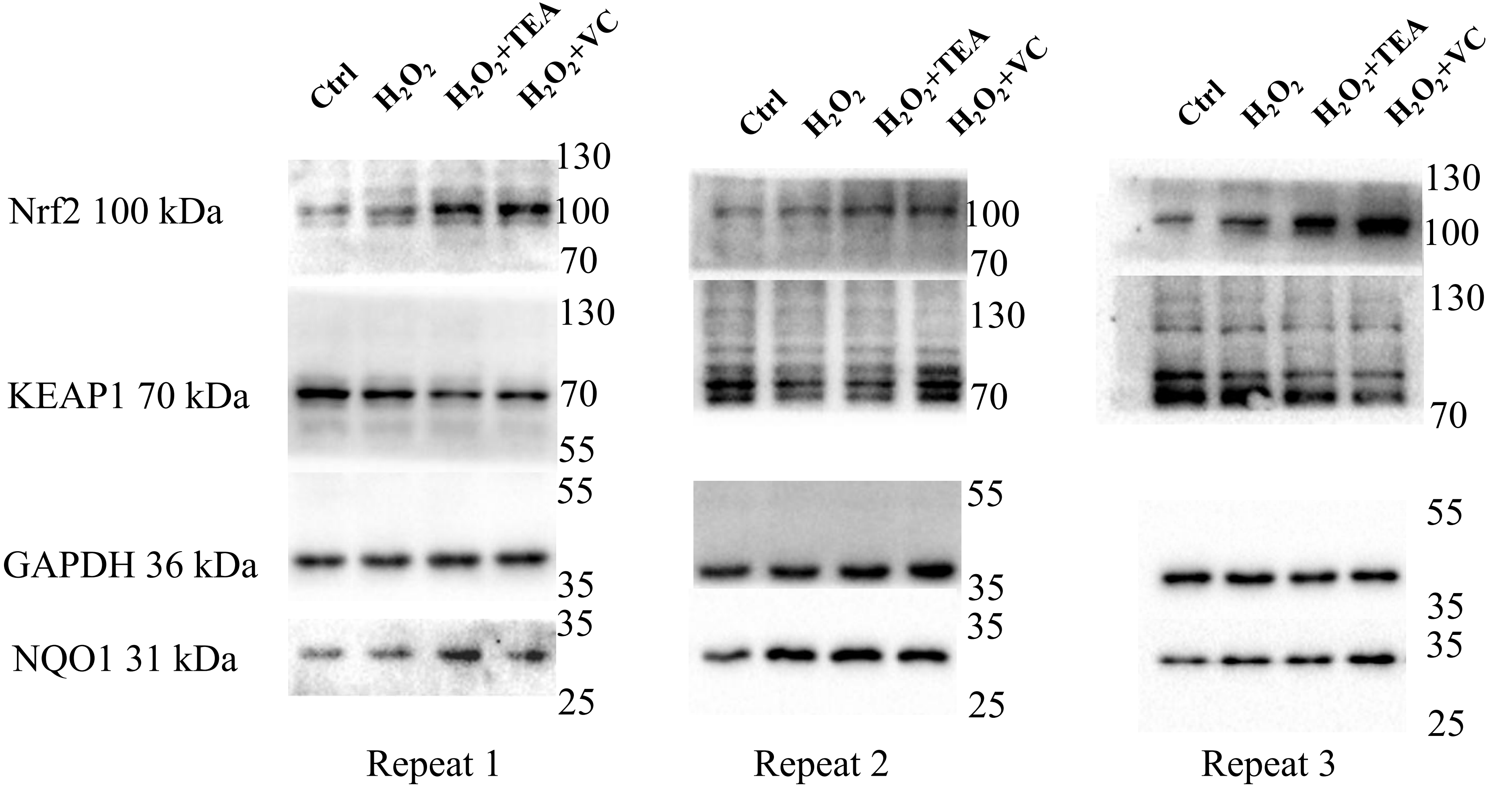

Figure S3. Three exposures of KEAP1, NQO1 and Nrf2 protein western blotting

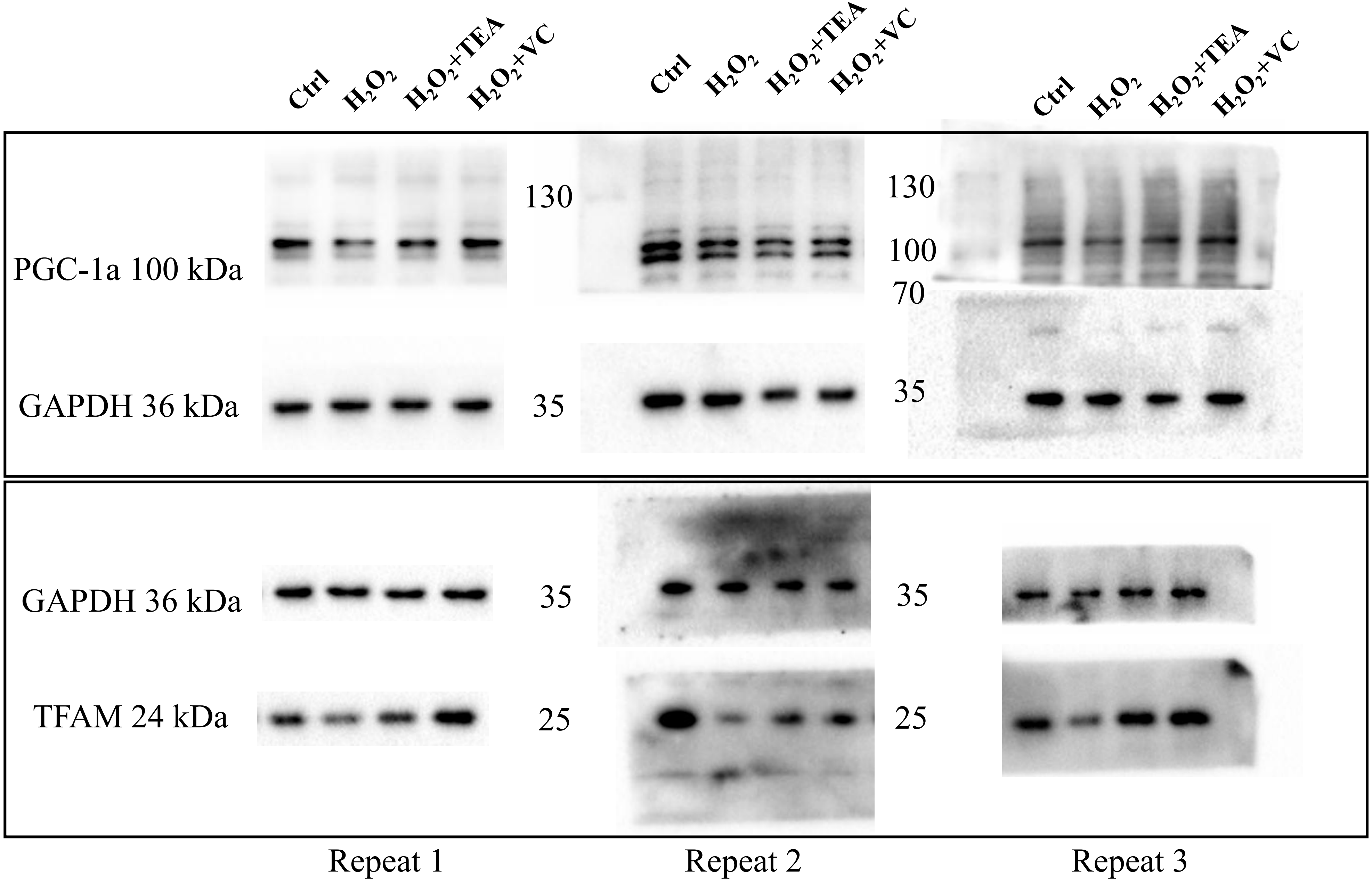

Figure S4. Three exposures of TFAM and PGC-1a protein western blotting

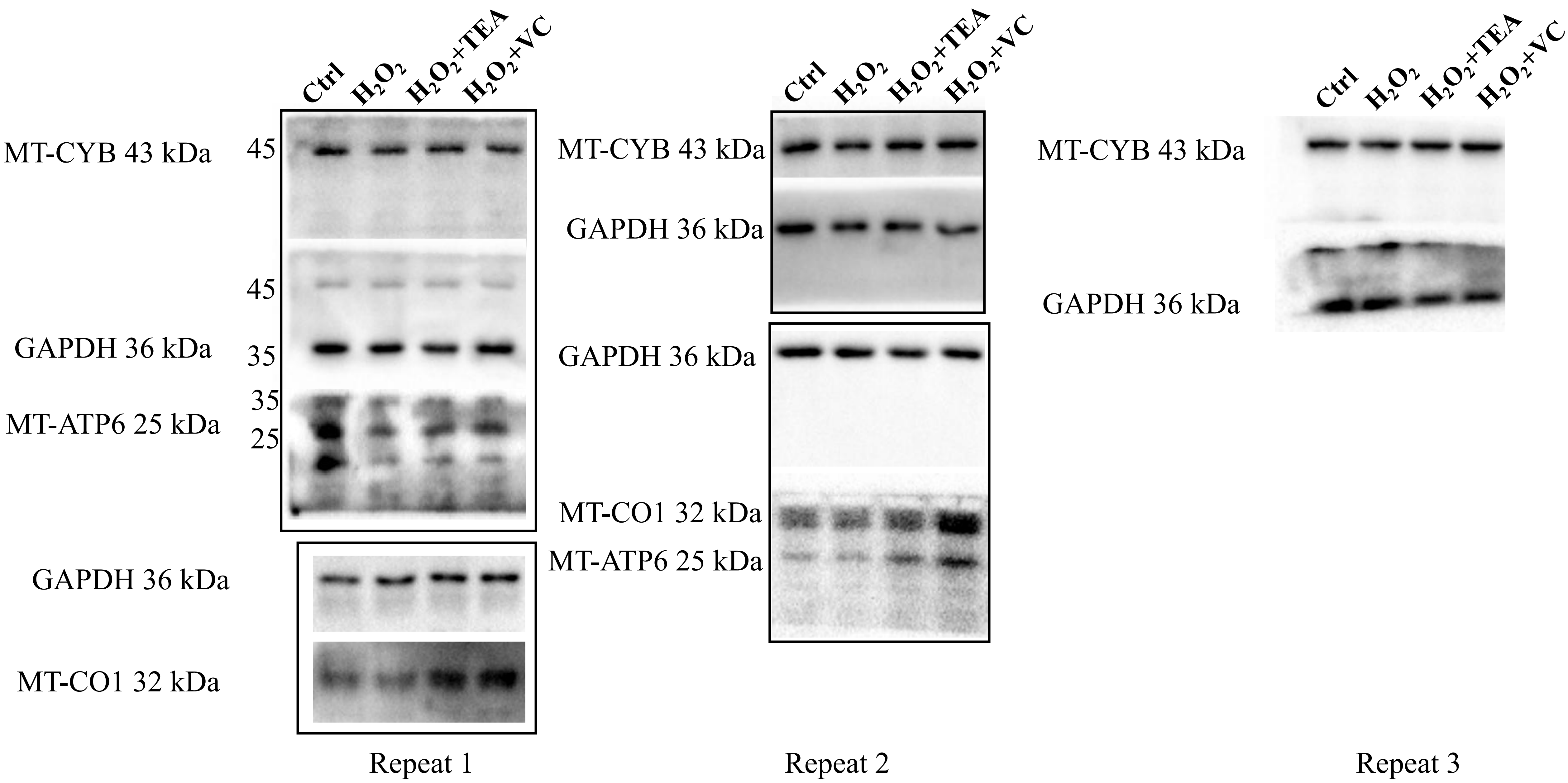

Figure S5-1. Three exposures of MT-CYB, MT-CO1 and MT-ATP6 protein western blotting

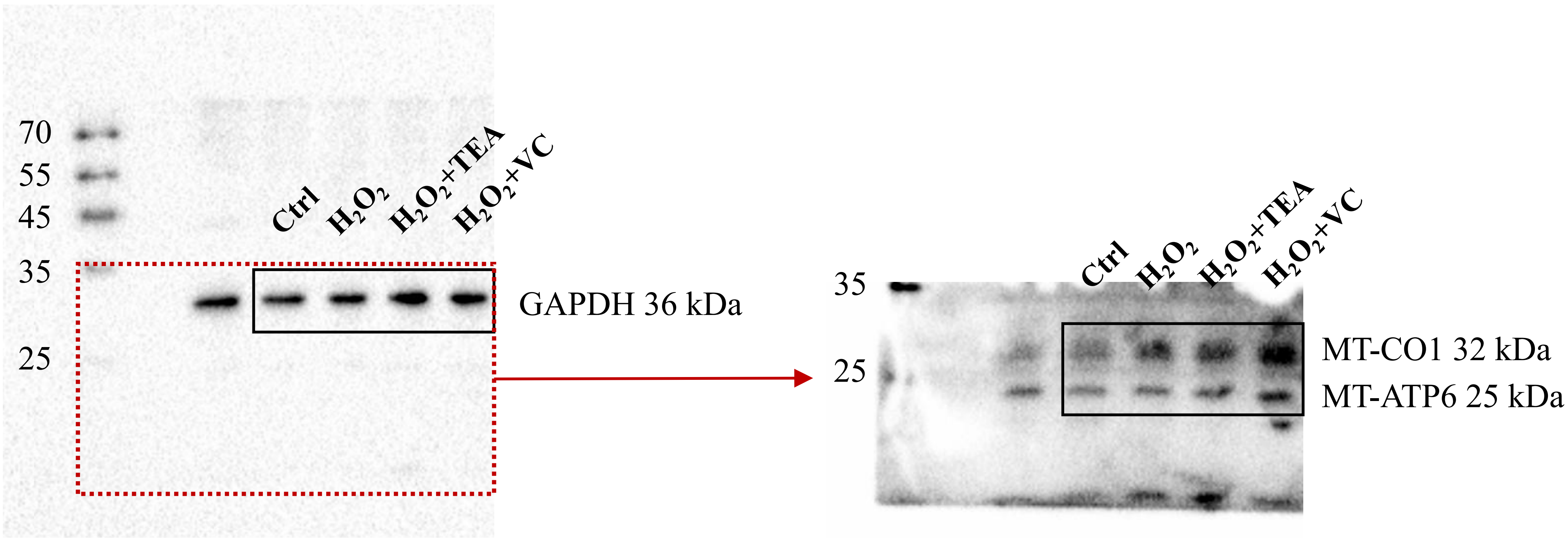

Repeat 3

Figure S5-2. Three exposures of MT-CYB, MT-CO1 and MT-ATP6 protein western blotting

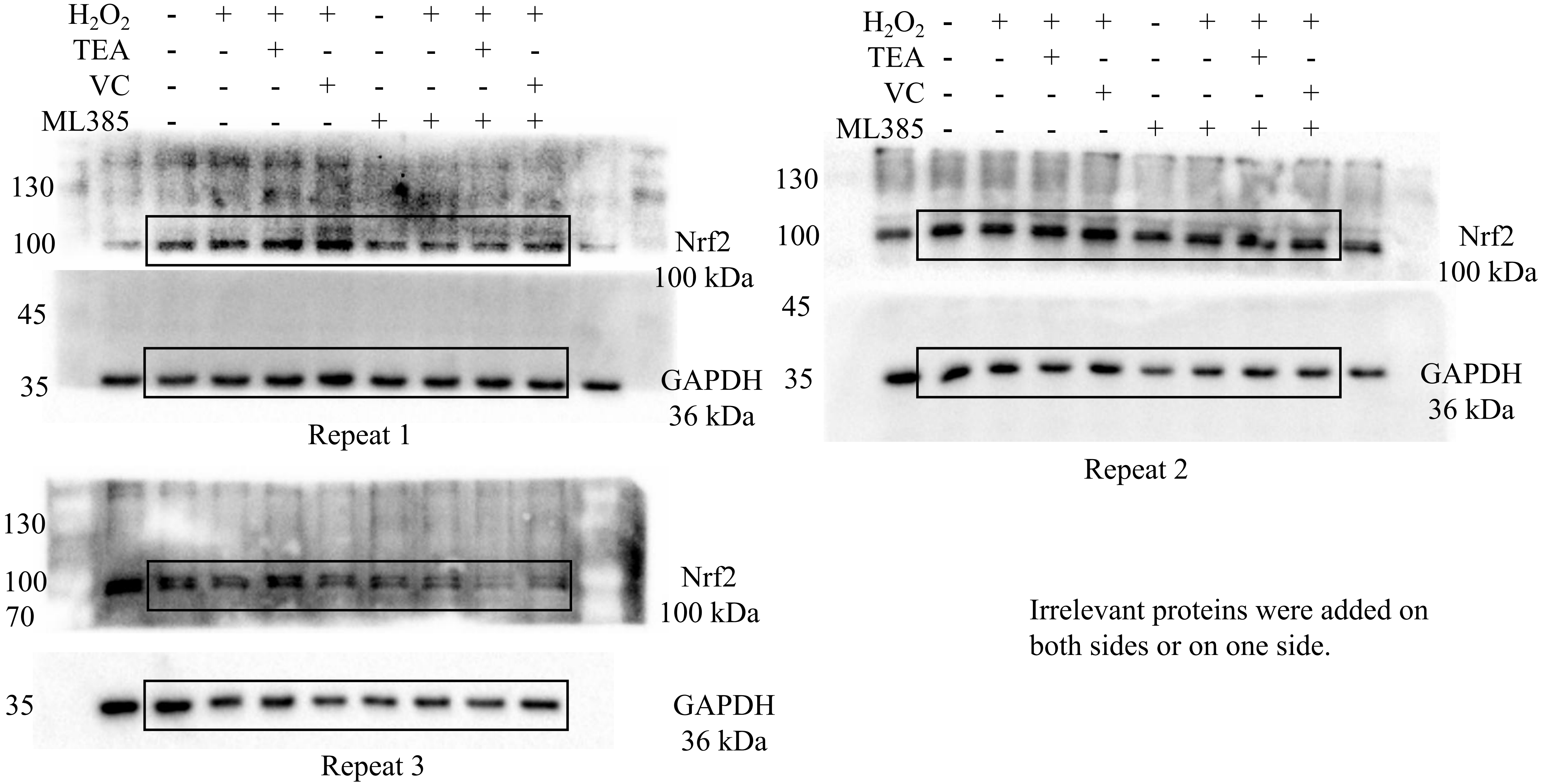

Figure S6. Three exposures of Nrf2 protein western blotting

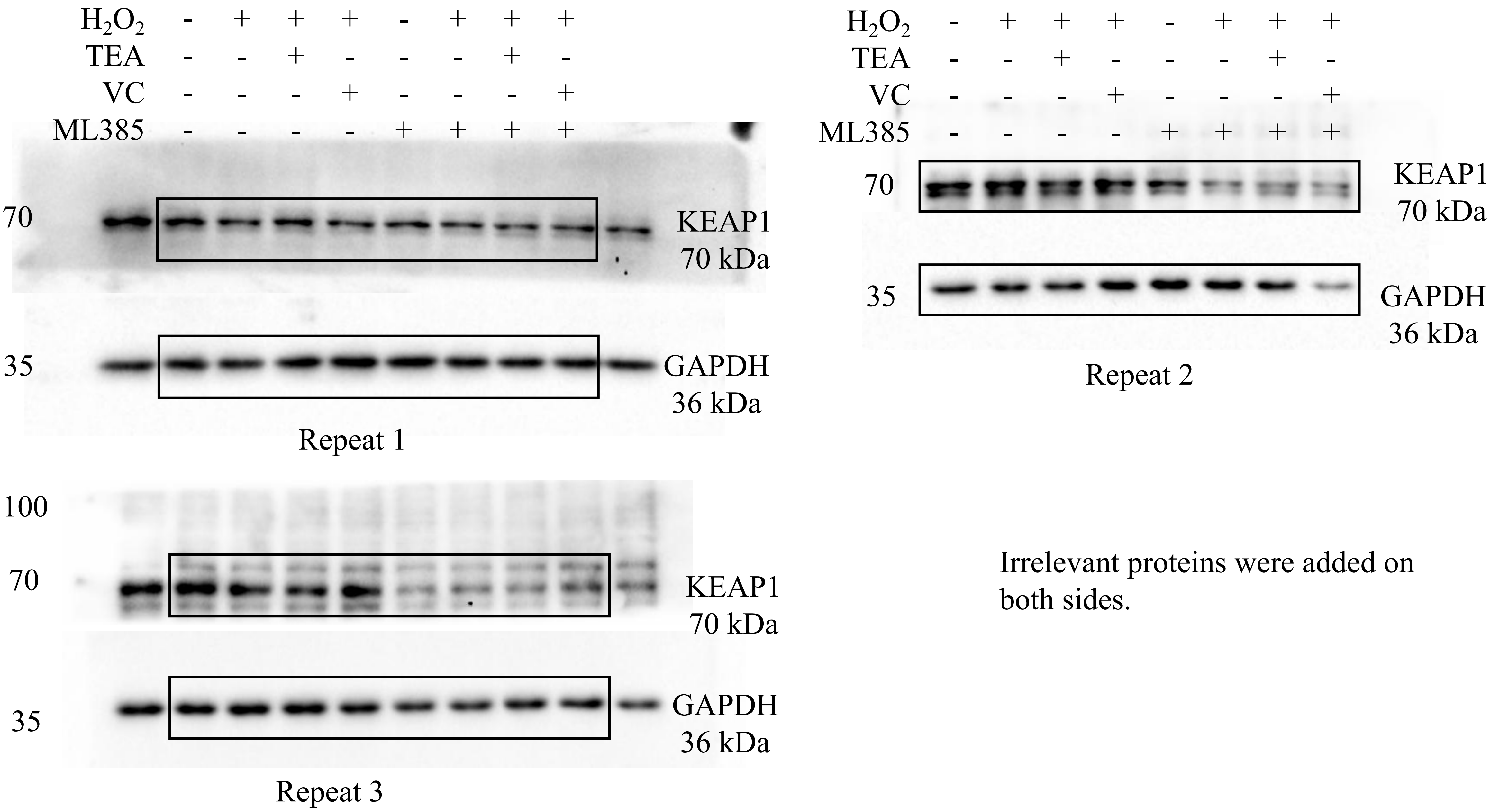

Figure S7. Three exposures of KEAP1 protein western blotting

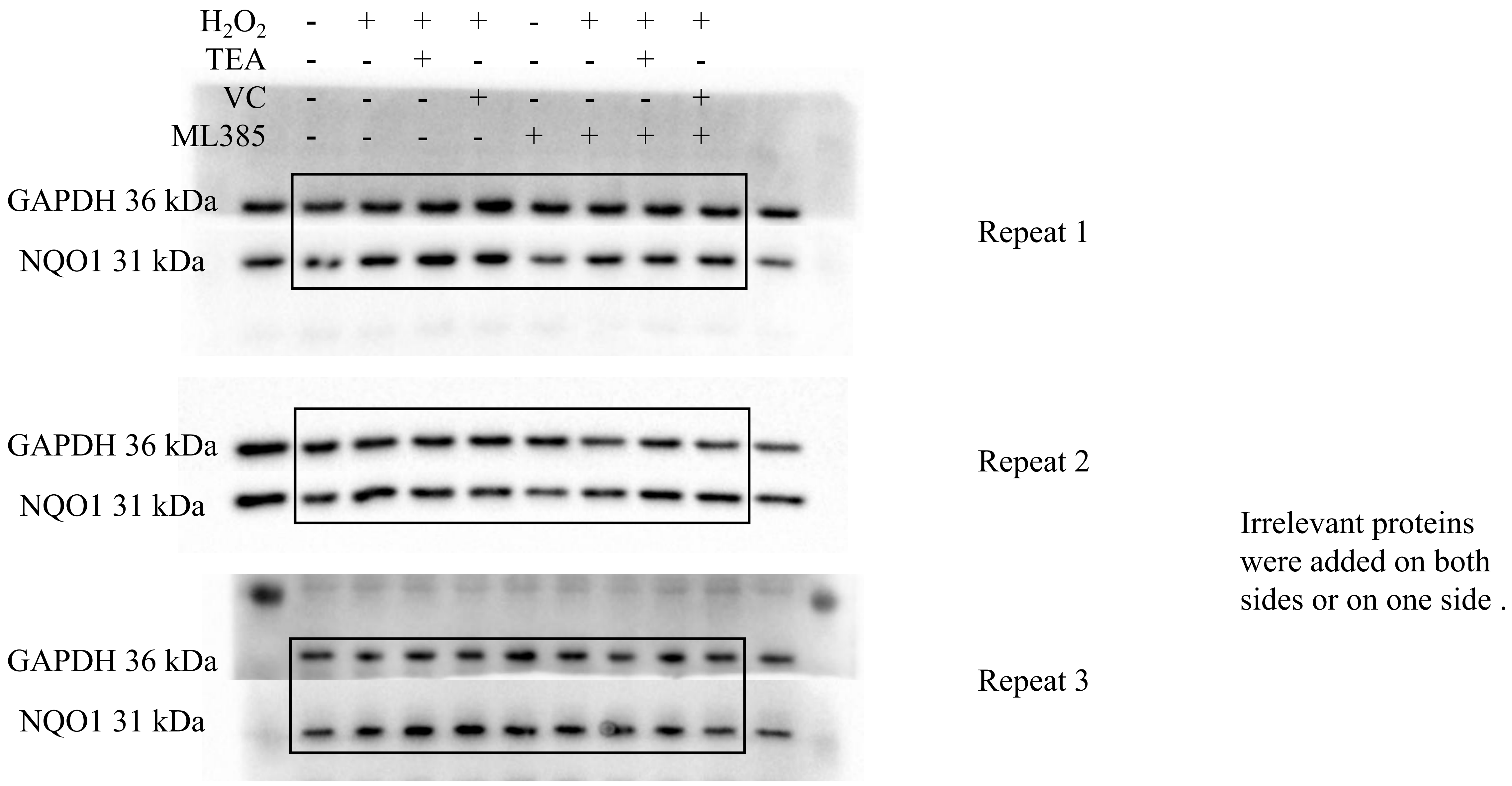

Figure S8. Three exposures of NQO1 protein western blotting

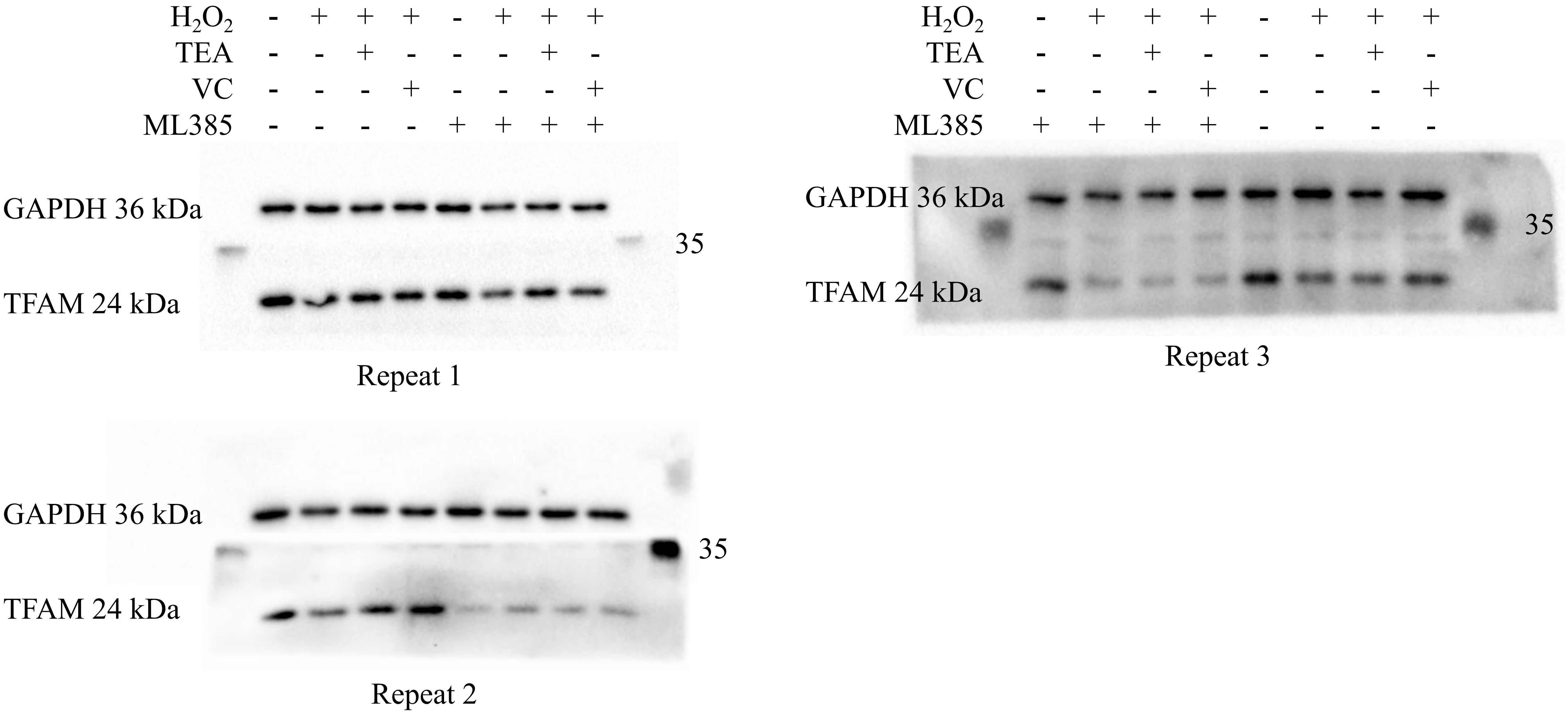

Figure S9. Three exposures of TFAM protein western blotting

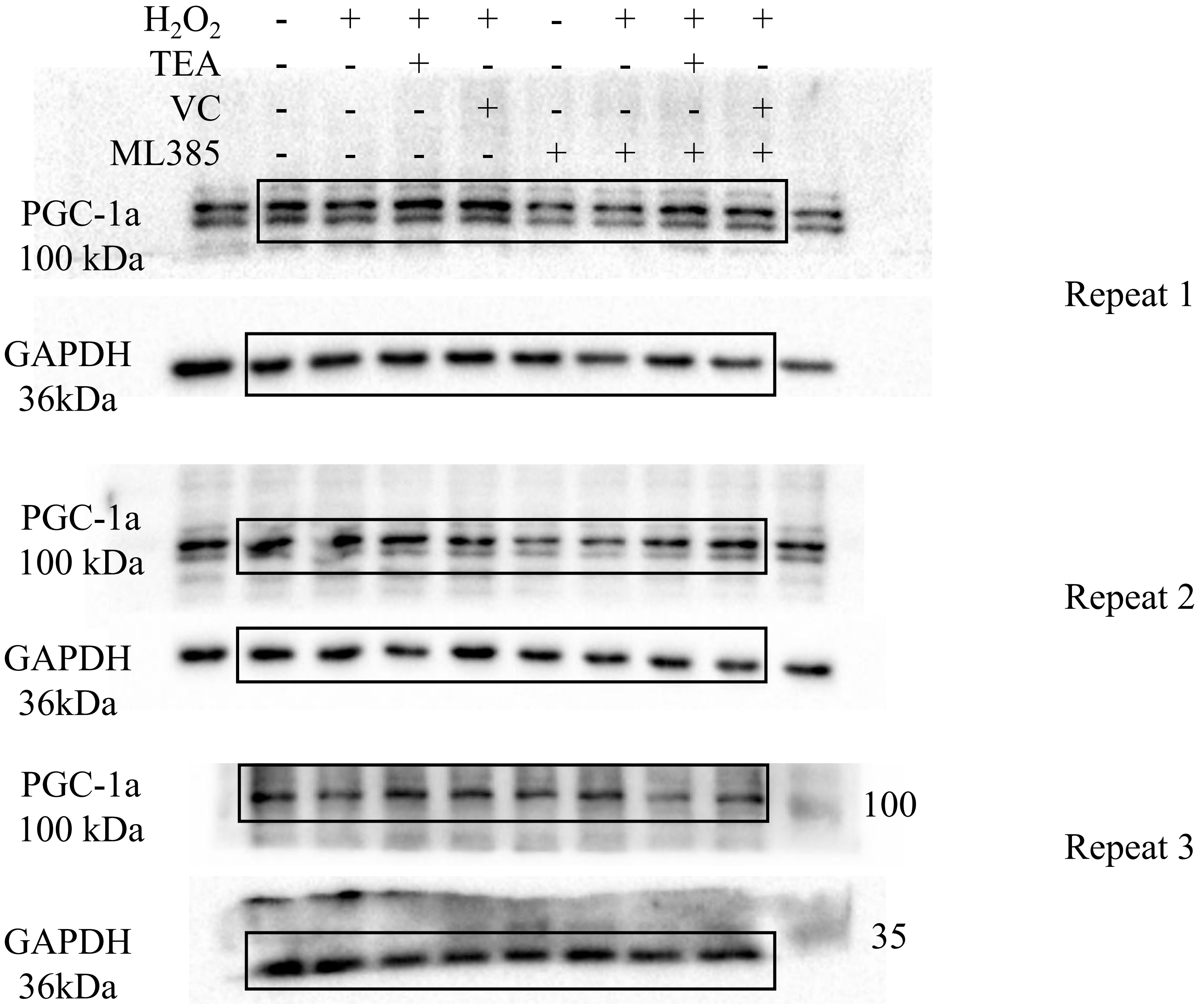

Irrelevant proteins were added on both sides.

Figure S10. Three exposures of PGC-1a protein western blotting

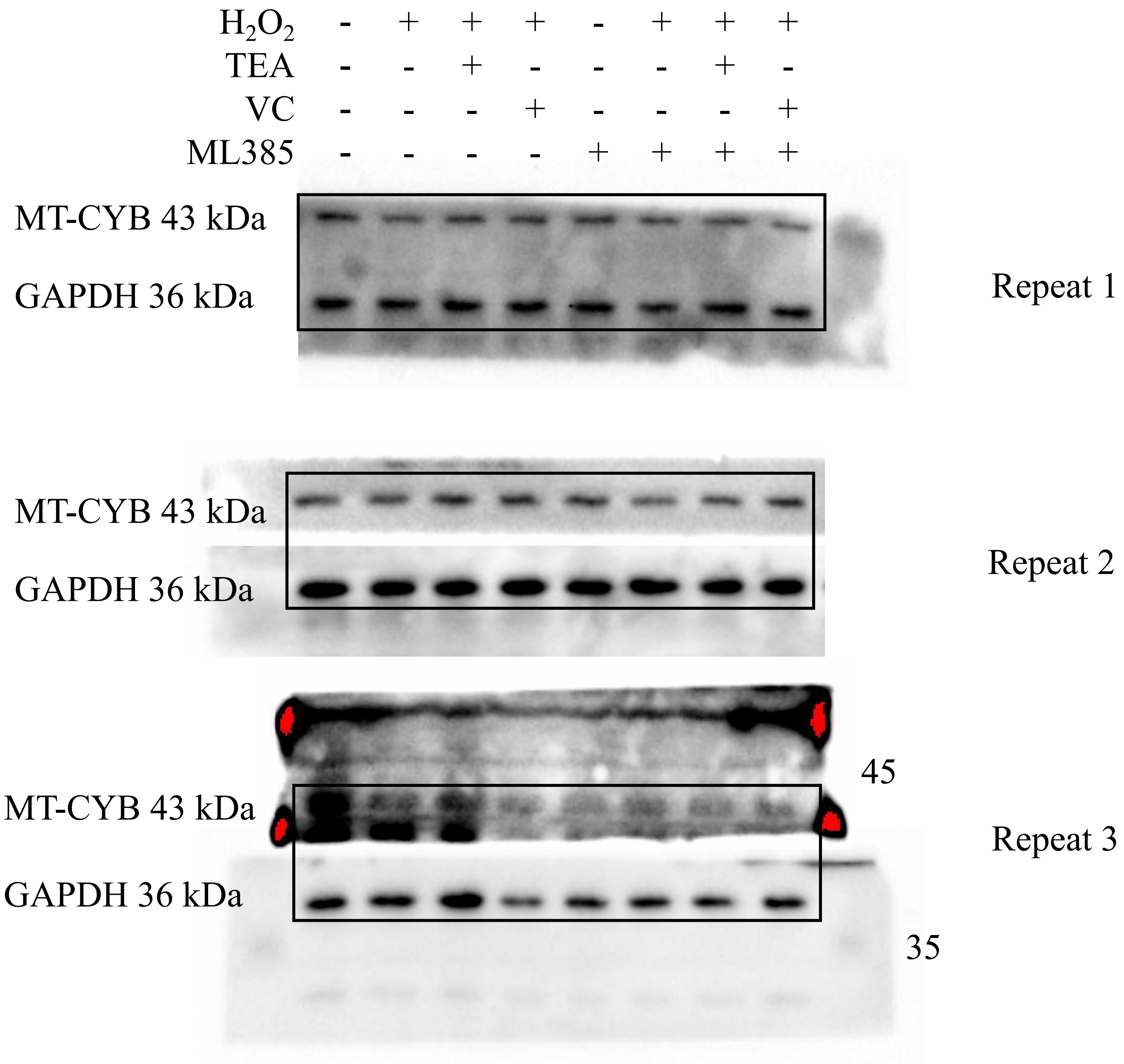

Figure S11. Three exposures of MT-CYB protein western blotting

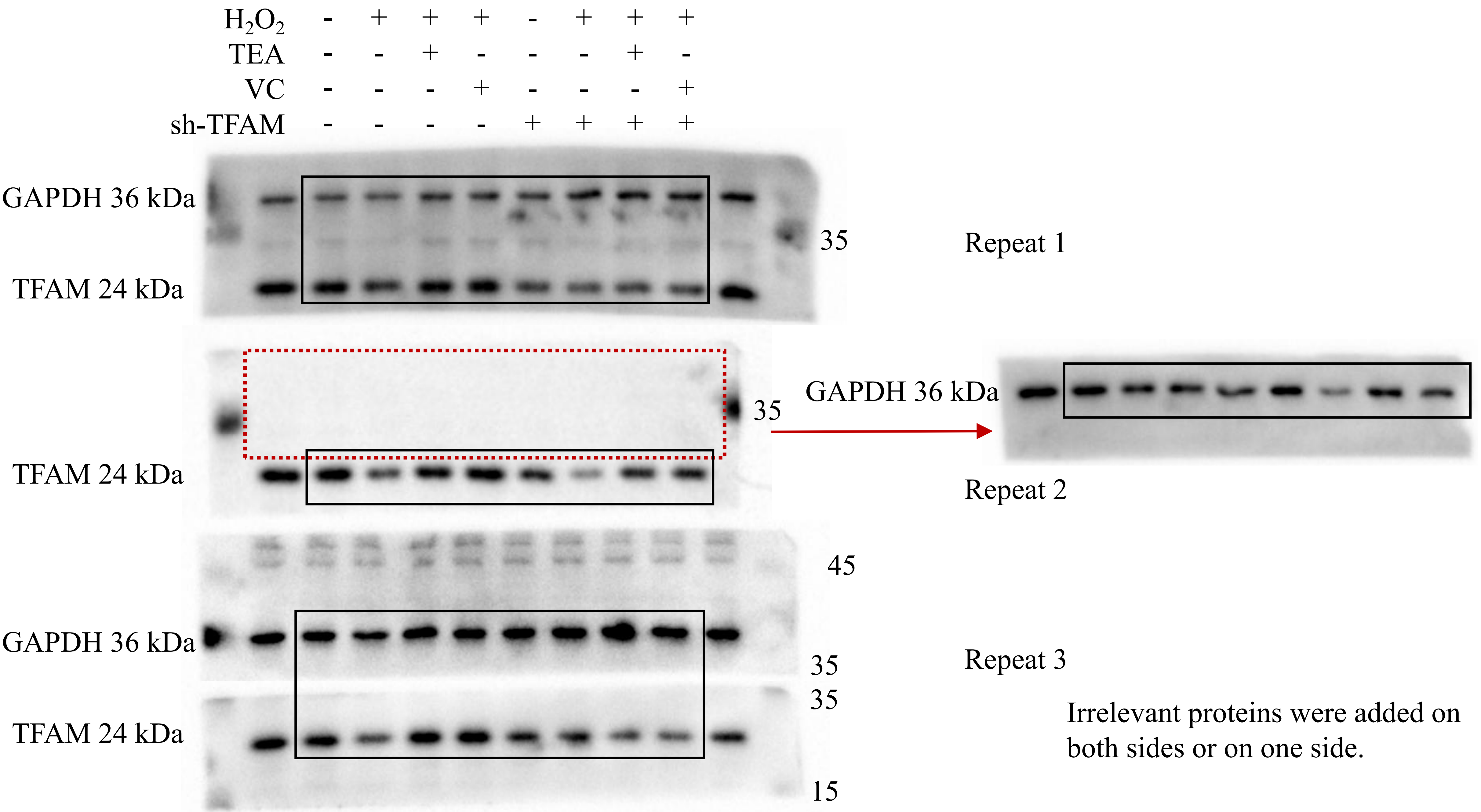

Figure S12. Three exposures of TFAM protein western blotting

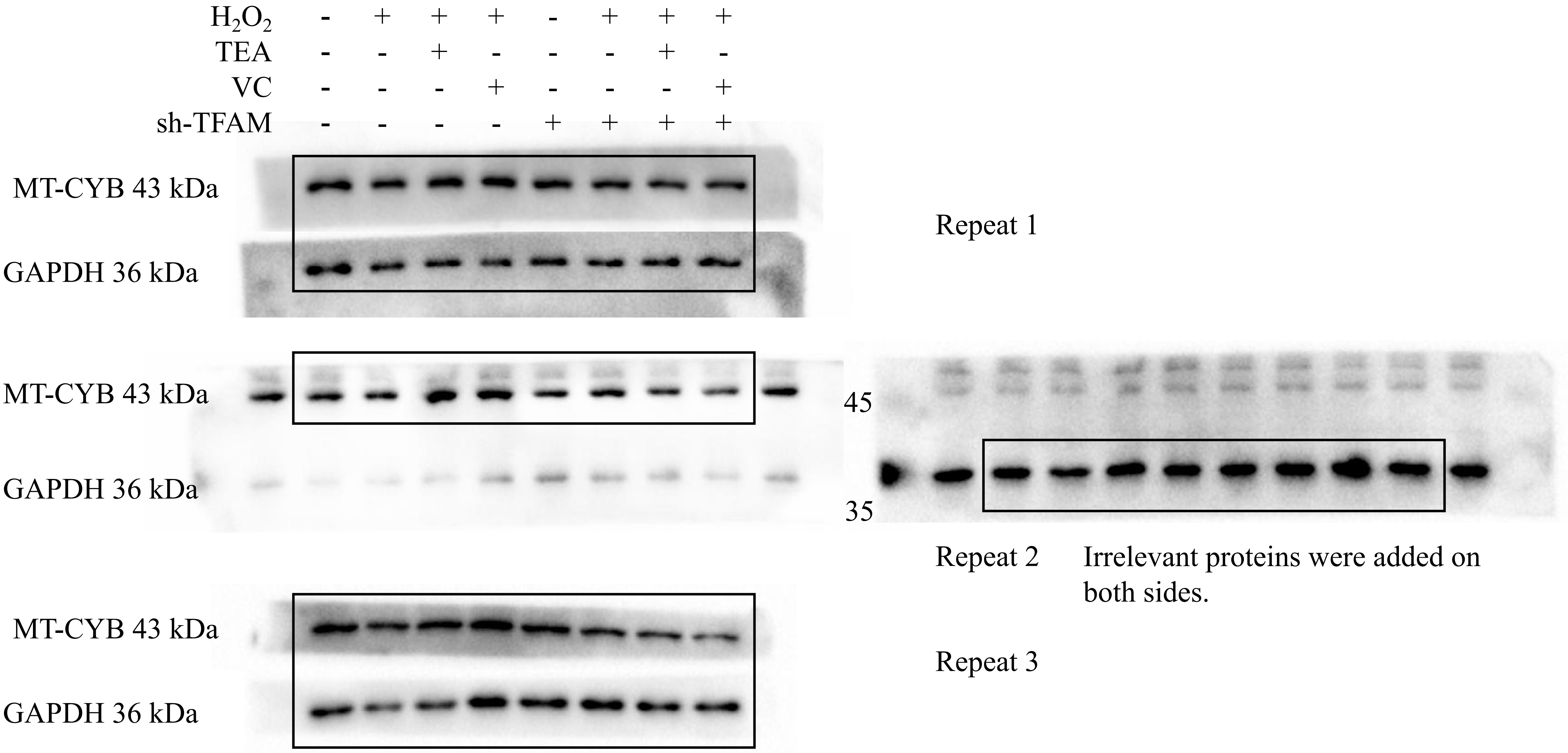

Figure S13. Three exposures of MT-CYB protein western blotting
